# Predictors of survival and neurologic outcome for adults with extracorporeal cardiopulmonary resuscitation: A systemic review and meta-analysis

**DOI:** 10.1097/MD.0000000000013257

**Published:** 2018-11-30

**Authors:** Junhong Wang, Qingbian Ma, Hua Zhang, Shaoyu Liu, Yaan Zheng

**Affiliations:** a Emergency Department, Peking University Third Hospital; b Research Center of Clinical Epidemiology, Peking University Third Hospital, Beijing, China.

**Keywords:** adult extracorporeal cardiopulmonary resuscitation, neurologic outcome, predictors, survival, systemic review and meta-analysis

## Abstract

**Background:**

This systemic review aimed to explore the predictors of discharge and neurologic outcome of adult extracorporeal cardiopulmonary resuscitation (ECPR) to provide references for patient selection.

**Methods:**

Electronically searching of the Pubmed, Embase, Cochrane Library, and manual retrieval were done for clinical trials about predictors for adult ECPR which were published between January 2000 and January 2018 and included predictors for discharge and neurologic outcome. The literature was screened according to inclusion and exclusion criteria, the baseline information and interested outcomes were extracted. Two reviewers assessed the methodologic quality of the included studies and the quality of evidence for summary estimates independently. Pooled mean difference (MD) or odds ratio (OR) and 95% confidence interval (CI) were calculated by Review Manager Software 5.3. At last the quality of evidence for summary estimates was appraised according to Grading of Recommendations Assessment, Development, and Evaluation rating system.

**Results:**

In 16 studies, 1162 patients were enrolled. Out-of-hospital cardiac arrest (CA) (OR 0.58, 95% CI 0.36–0.93, *P* = .02), in-hospital CA (OR 1.73, 95% CI 1.08–2.77, *P* = .02), witnessed CA (OR 5.2, 95% CI 1.18–22.88, *P* = .01), bystander cardiopulmonary resuscitation (CPR) (OR 7.35, 95% CI 2.32–23.25, *P* < .01), initial shockable rhythm (OR 2.29, 95% CI 1.53–3.42, *P* < .01), 1st recorded nonshockable rhythm (OR 0.44, 95% CI 0.29–0.66, *P* < .01), CPR duration (MD −13.84 minutes, 95% CI −21 to −6.69, *P* < .0001), arrest-to-extracorporeal membrane oxygenation (ECMO) (MD −17.88 minutes, 95% CI −23.59 to −12.17, *P* < .01), PH (MD 0.14, 95% CI 0.08–0.21, *P* < .01), lactate (MD −3.66 mmol/L, 95% CI −7.15 to −0.17, *P* = .04), and percutaneous coronary intervention (PCI) (OR 1.63, 95% CI 1.02–2.58, *P* = .04)were identified as the survival predictors of ECPR. Shockable rhythm (OR 2.33, 95% CI 1.20–4.52, *P* = .01) and CPR duration (MD −9.85 minutes, 95% CI −15.71 to −3.99, *P* = .001) were identified as the neurologic outcome predictors of ECPR.

**Conclusion:**

Current evidence showed that in-hospital CA, witnessed CA, bystander CPR, initial shockable rhythm, shorter CPR duration and arrest-to-ECMO duration, higher baseline PH, lower baseline lactate and PCI were favourable survival predictors of adult ECPR, and shockable rhythm and shorter CPR duration were good neurological outcome predictors of adult ECPR.

## Abstract 2

Supplemental Digital Content is available in the text

## 1 Introduction

Although cardiopulmonary resuscitation (CPR) has been used widely for treatment of cardiac arrest (CA), it provides only 30% to 40% of normal blood flow to the brain even when delivered according to guidelines.^[1]^ Both in-hospital CA (IHCA) and out-of-hospital CA (OHCA) patients are associated with a poor prognosis.^[2,3]^ The increased social and economic burdens for CA make their treatment and recovery a major public health issue.

Extracorporeal cardiopulmonary resuscitation (ECPR) by means of venous-atrial extracorporeal membrane oxygenation (ECMO) was effective to restore circulation and provide oxygen for refractory CA. The mortality and neurologic function recovery of ECPR recipient were more satisfactory than that of CPR recipients.^[4]^ The American Heart Association pointed out that in settings where it can be rapidly implemented, ECPR may be considered for select patients with CA if some conditions were met.^[5]^

However, the potential benefit of ECPR should be balanced against the risk of futility, physical disability and psychologic disorder of survivals, and high cost of the technique. There was no consensus about the criteria for starting ECPR. Identifying survival and neurologic predictors associated with ECPR may help physician predict clinical outcomes, improve discretion of treating physician, and further improvements in the efficiency of ECPR use. Previous systemic reviews or meta-analyses discussed the survival predictors of ECPR for IHCA and OHCA separately or the quality of evidence across studies is very low if they included both IHCA and OHCA; however, surprisingly, some of the results of these studies are similar.^[6–10]^ Different from IHCA, the data for OHCA were relatively scarce and conflicting. One of the explanations for the variability of the results was the duration from CA to ECMO initiation.^[11]^ The main reason for different prognoses between IHCA and OHCA with ECPR might be the delay of ECPR rather than the location of CA.^[12]^ On the basis of these findings, a systemic review and meta-analysis was performed to evaluate the prognostic significance of prespecified baseline characteristics in terms of survival and neurologic outcome for ECPR recipients suffering IHCA or OHCA.

## 2 Materials and methods

This meta-analysis was performed according to the Cochrane Handbook for Systematic Reviews of Interventions (version 5.1.0) and presented based on Preferred Reporting Items for Systemic Reviews and Meta-analyses Guidelines.^[13]^ PRISMA checklist shows this in more detail. The protocol for this article is available in PROSPERO (CRD42018086774). As all analyses were based on previous published studies, no ethical approval and patient consent were required.

### 2.1 Data source and search strategy

Potentially relevant studies were identified and screened for retrieval by a thesaurus search. PubMed, Embase, and the Cochrane Library were electronically searched for relevant citations using individualized search strategies prepared for each database by 2 independent researchers. Moreover magazines and meeting abstracts in our hospital library were manually retrieved. The search terms included both standardized medical subject heading (Mesh) and text words (see Supplemental file 1, which illustrates the search strategies). Original clinical trials were searched. There were no language restrictions and all searched studies were published between January 2000 and January 2018. No document restrictions and no methodology filters were applied. The search was limited to humans.

### 2.2 Inclusion and exclusion criteria

Trials were selected based on the following criteria: trails enrolling adults suffered from IHCA or OHCA; trails providing predictor data; the endpoint included survival from the hospital or the neurologic outcome at discharge; and randomized control trials, clinical trials, case–control trials or cohort trials.

Exclusion criteria were: the repetition of published literature; animal experiments or trails including pediatric patients; venous-atrial ECMO used for cardiogenic shock; endpoint did not include survival from the hospital or the neurologic outcome at discharge; venous-atrial ECMO used after surgery; unlike previous meta-analysis including studies which employed extracorporeal bypass as the extracorporeal circulation, such studies was excluded, since the implementation process and indication of the former was quite different from the latter; studies recruiting fewer than 10 participants; and case series.

### 2.3 Assessment of methodologic quality

Two reviewers independently assessed the methodologic qualities for each study using the Newcastle–Ottawa Quality Assessment scale (NOS)^[14]^ for case–control studies or cohort studies. Any unresolved disagreements between reviewers were resolved by consensus.

### 2.4 Data extraction

Two reviewers extracted the following information from each study independently, using a standard form: lead author; publication year, country of origin, enrolment period, number of study sites, etiology of CA, participant characteristics, and study design. Disagreements were reconciled through discussion. Cerebral performance category (CPC) 1 was defined as good cerebral performance and CPC 2 was defined as moderate cerebral disability. CPC 1 and 2 was deemed as good neurologic recovery, while CPC 3 to 5 was regarded as bad neurologic recovery.

The prespecified predictors of interest included patient age and gender, body mass index (BMI), population of OHCA/IHCA, witnessed CA, bystander CPR, initial shockable rhythm, initial nonshockable rhythm, CPR duration, arrest-to-ECMO duration, baseline lactate concentration and arterial PH, whether percutaneous coronary intervention (PCI) carried out subsequently, and if there was restoration of spontaneous circulation (ROSC) before ECMO implementation. According to the Utstein style, bystander CPR was defined as an attempt to perform CPR by someone who was not part of an organized emergency response system.^[15]^ The initial rhythm was defined as the first recorded rhythm. Shockable rhythm included pulseless ventricular tachycardia and ventricular fibrillation, while nonshockable rhythm included pulseless electricity activity and asystole. Baseline lactate and arterial PH were measured after patients arrived at hospital. Definition of sustained ROSC was continuous maintenance of spontaneous circulation for ≥20 minutes. CPR duration was the primary outcome, because it was remained controversial in vast majority of studies and can be optimized by treating physicians to improve outcome.

### 2.5 Statistical analysis

Meta-analysis was conducted by Review Manager Software 5.3 (The Cochrane Collaboration, Oxford, UK). Factors documented in at least 3 studies were entered into a meta-analysis. Mean and standard deviation values were computed according to Wan et al for primary studies reporting median baseline values and interquartile range.^[16]^ Pooled means and standard deviations or possibilities were calculated according to Jin et al, respectively, for continuous variables.^[17]^ The heterogeneity of pooled data was estimated by calculating the *Q* and *I*^2^, and it was regarded as significant when *I*^2^ ≥ 50% or *P* < .05. Possible reasons for heterogeneity of primary outcome were investigated by subgroup analyses. Pooled mean difference (MD) or odds ratio (OR) was calculated, respectively, for continuous variables or categorical variables between outcome groups. *P* < .05 was considered statistically significant. Publication bias was estimated by the visual inspection of funnel plot if studies included were ten or more. We conducted sensitivity analysis by leaving out negative direction study to assess the robustness of the findings.

The quality of evidence for summary estimates was assessed by 2 reviewers independently according to the Grading of Recommendations Assessment, Development, and Evaluation (GRADE) rating system based on study design, limitations, indirectness of evidence, inconsistency in results across studies, imprecision in summary estimates, and likelihood of publication bias.^[18]^ Imprecision was graded into 3 levels by the following 2 criteria: either the lower or the upper bound of the confidence intervals (CIs) was less or more than 20% of the point estimate, respectively; 300 events for dichotomous outcomes or 400 participants for continuous outcomes. Once a criterion was not met, we down 1 point. The remaining items were graded into 2 levels.

## 3 Results

### 3.1 Identification of studies

Electronical searches of the databases yielded 5027 related articles and 28 articles were retrieved through manual searches. These articles were screened by excluding duplicates at first and 3139 records were identified. Titles and abstracts of these records were screened for inclusion. Eighty-three trials were considered for full-text screening. Among them, 67 were excluded because they did not fulfill inclusion criteria. These studies and reasons for their exclusion are listed in the Supplemental file 2. Sixteen trials^[19–34]^ were ultimately included in this meta-analysis. The flowchart of systemic review was displayed in PRISMA Flow Diagram.

### 3.2 Study characteristics and quality of the included studies

Table 1 displayed characteristics and variables of included studies. These studies were published from 2008 to 2018, while patient enrollment periods extended from 1994 to 2016. Those included were all high-grade articles according to the NOS (see Supplemental file 3, which illustrates the details of NOS). Fifteen retrospective studies and 1 prospective study reported the outcome of overall 1162 patients. The neurologic outcome of 87.6% survivors achieved CPC1 and 2. Fourteen articles^[19–26,28–30,32–34]^ investigated the predictors associated with survival to discharge, while 3 articles^[20,27,31]^ investigated the predictors associated with neurologic outcome at discharge. In 5 trials, we extracted low-flow duration as CPR duration,^[19–23]^ because the former was defined as the time from CPR initiation to ECPR.

**Table 1:**
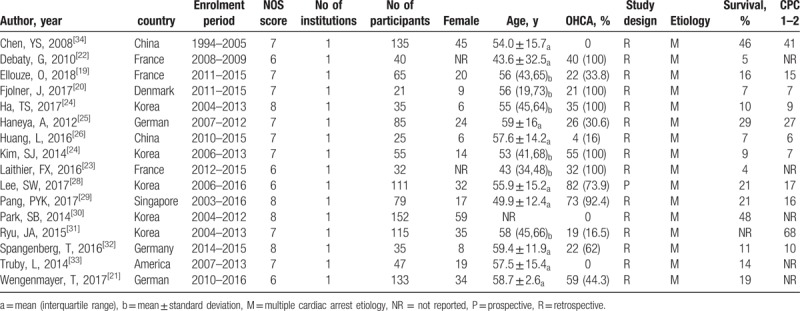
Characteristics of included studies.

### 3.3 Outcome

#### 3.3.1 Predictors of survival

##### 3.3.1.1 Variables of patients’ baseline information

There was no significant difference in between 163 survivors and 432 nonsurvivors in terms of age ([54.5 ± 15.9] years vs [56.7 ± 16.7] years, MD −3.83 [−8.65, 0.99] years, *P* = .12) (Table 2, see Supplemental file 4A, which illustrates forest plot of age in terms of survival predictor). No statistical difference was found between 168 survivors and 422 nonsurvivors in terms of male (70.8% vs 69.0%, OR 1.12 [0.75, 1.67], *P* = .57) (Table 2, see Supplemental file 4B, which illustrates forest plot of male in terms of survival predictor). No significant difference was found between 98 survivors and 211 non-survivors in terms of BMI [(25.5 ± 5.0) kg/m^2^ vs (26.2 ± 6.4) kg/m^2^; MD −0.24 (−1.23, 0.74) kg/m^2^, *p* = 0.63] (see Supplemental file 4C, which illustrates forest plot of BMI in terms of survival predictor).

**Table 2:**
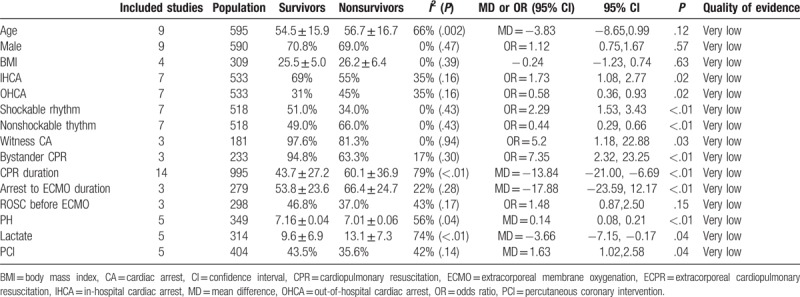
Pooled mean difference or pooled odds ratio of predictors for survival.

##### 3.3.1.2 Variables of CA

Seven researches were included in the analysis of OHCA and IHCA encompassing 533 patients. The odds of IHCA tended to be higher [69.4% vs 55.3%, OR 1.73 (1.08, 2.77), 95%CI, *P* = 0.02] while the odds of OHCA tended to be lower [OHCA 31% vs 45%, OR = 0.58 (0.36, 0.93), *P* = 0.02] in survivors than non-survivors (Table 2, see Supplemental file 4D, E, which illustrates forest plot of OHCA or IHCA in terms of survival predictor separately). For both predictors, the results were rather unchanged in sensitivity analyses (Table 2, see Supplemental file 4F, G, which illustrates sensitivity analysis of OHCA or IHCA in terms of survival predictor separately).

The odds of initial shockable rhythm tended to be higher in 147 survivors than 371 nonsurvivors (51.0% vs 34.0%, OR 2.29 [1.53, 3.43], *P* < .01), while survivors had a significantly lower likelihood of having a nonshockable rhythm than nonsurvivors (49.0% vs 66.0%, OR = 0.44 [0.29, 0.66], *P* < .01) (Table 2, see Supplemental file 4H, I, which illustrates forest plot of shockable rhythm or nonshockable rhythm in terms of survival predictor separately). Sensitivity analyses indicated that above results were robust (see Supplemental file 4J, K, which illustrates sensitivity analysis of shockable rhythm or nonshockable rhythm in terms of survival predictor separately).

##### 3.3.1.3 Variables of CPR

The pooled OR in witness CA was 5.2 (95% CI 1.18 to 22.88; *P* = 0.03) and a significant difference in bystander CPR was found [OR7.35 (2.32, 23.25), *P* < 0.01] favoring 42 survivors to 139 non-survivors enrolled in 3 articles (Table 2, see Supplemental file 4L, M, which illustrates forest plot of witnessed CA or bystander CPR in terms of survival predictor separately).

**Figure 1:**
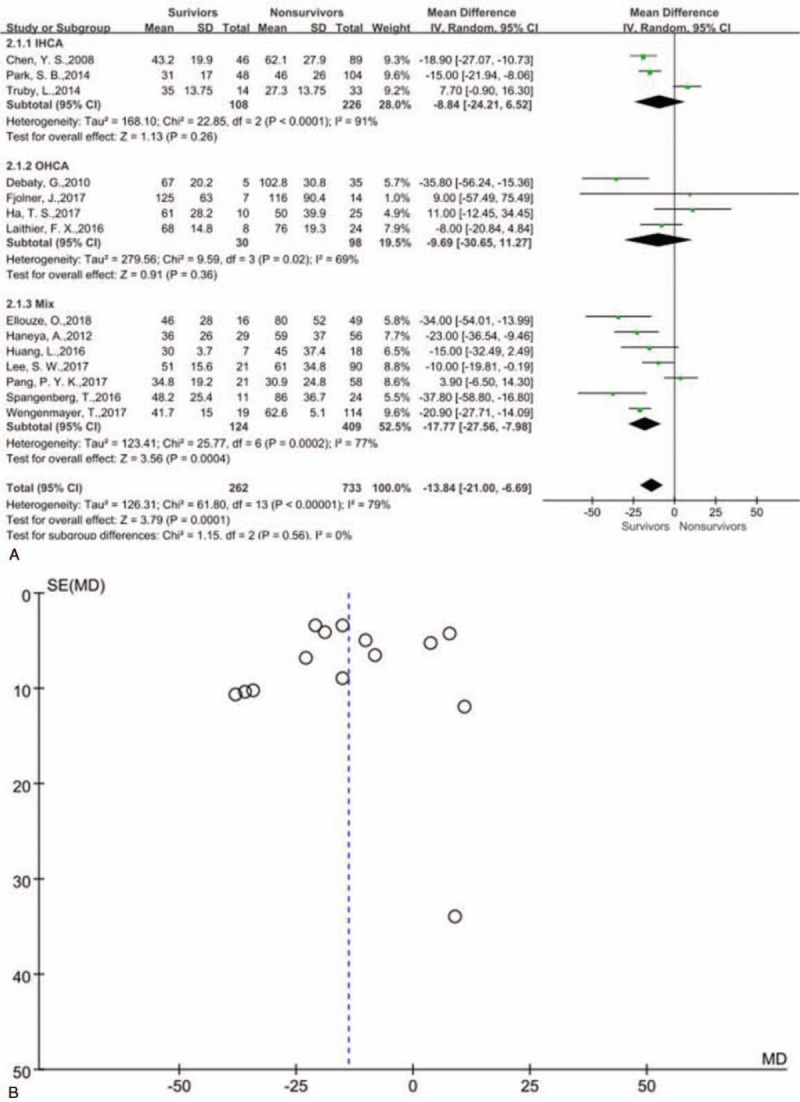
(A) Forest plot of cardiopulmonary resuscitation duration in terms of survival predictor and subgroup analysis. (B) Funnel plot of cardiopulmonary resuscitation duration in terms of survival predictor.

Overall CPR duration tended to be shorter in 262 survivors than 733 nonsurvivors [(43.7 ± 27.2) minutes vs (60.1 ± 36.9) minutes, MD −13.84 (−21.00, −6.69) minutes, *P* < .01] (see Fig. 1A, which illustrates forest plot of CPR duration in terms of survival predictor); however, substantial between-study heterogeneity was observed for this analysis (*P* < .01, *I*^2^ = 79%). There was publication bias (Fig. 1B), and a sensitivity analysis was taken and indicated that this outcome was robust (*P* < .01) (Table 2, see Supplemental file 4N, which illustrates sensitivity analysis of CPR duration in terms of survival predictor). We divided the included studies into 3 groups as OHCA, IHCA, and mix location of CA, yet the location of CA could not interpret the between-study heterogeneity (Fig. 1A, which illustrates subgroup analysis of CPR duration in terms of survival predictor).

##### 3.3.1.4 Variables of ECMO

The pooled MD in arrest-to-ECMO duration was −17.88 minutes (95% CI [−23.59 to −12.17] minutes, *P* < .01) for 50 survivors relative to 229 nonsurvivors ([53.8 ± 23.6] minutes vs [66.4 ± 24.7] minutes) enrolled in 3 primary studies (Table 2, see Supplemental file 4O, which illustrates forest plot of arrest-to-ECMO duration in terms of survival predictor).

There was no significant difference of ROSC before ECMO between 79 survivors and 219 non-survivors [46.8% vs 37.0%, OR 1.48 (0.87 to 2.50), *P* = 0.15] (Table 2, see Supplemental file 4 P). This result was robust in sensitivity analysis (see Supplemental file 4Q, which illustrates forest plot of ROSC before ECMO in terms of survival predictor).

##### 3.3.1.5 Laboratory variables

There were statistical difference between survivors and nonsurvivors for baseline PH and arterial lactate (PH: 7.16 ± 0.04 vs 7.01 ± 0.06, MD 0.14 [0.08, 0.21], *P* < .01; lactate [9.6 ± 6.9] mmol/L vs [13.1 ± 7.3] mmol/L, MD = −3.66 (−7.15, −0.17) mmol/L, *P* = .04) (Table 2, see Supplemental file 4R, S, which illustrates forest plot of baseline PH and lactate in terms of survival predictor separately).

##### 3.3.1.6 Percutaneous coronary intervention

Significant pooled OR estimate of PCI was 1.63 (95% CI 1.02–2.58; *P* = .04) for 115 survivors relative to 289 nonsurvivors (43.5% vs 35.6%) enrolled in 5 primary studies (Table 2, see Supplemental file 4T). This finding was robust in sensitivity analysis (see Supplemental file 4U, which illustrate forest plot of PCI in terms of survival predictor).

##### 3.3.1.7 Quality of evidence

The quality of evidence supporting the prognostic value of all predictors was considered very low. We downgraded 3 points with male, IHCA, and initial nonshockable rhythm, 4 points with BMI, OHCA, bystander CPR, shockable rhythm, PCI, and ROSC before ECPR, and 5 points with age, witnessed CA, CPR duration, arrest-to-ECMO duration, and lactate (see Supplemental file 5, which illustrates quality of evidence assessment with survival predictors according to GRADE rating system).

#### 3.3.2 Predictors of neurologic outcome

##### 3.3.2.1 Variables of patients’ baseline information

There was no significant difference in between 83 patients with CPC 1 and 2 and 105 patients with CPC 3 to 5 in terms of age ([55.8 ± 8.1] years vs [56.9 ± 22.5] years, MD = −2.5 [−8.45, 3.45] years, *P* = .41) (Table 3, see Supplemental file 4V, which illustrates forest plot of age in terms of good neurologic outcome predictor) and sex of male (69.9% vs 70.5%, OR 1.07 [0.54, 2.13], *P* = .85) (Table 3, see Supplemental file 4W, which illustrates forest plot of male in terms of good neurologic outcome predictor).

**Table 3:**
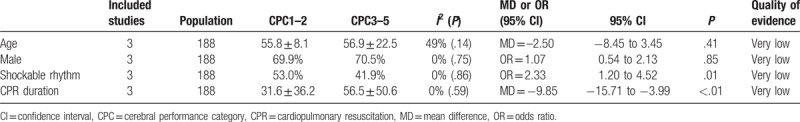
Pooled mean difference or pooled odds ratio of predictors for neurologic outcomes.

##### 3.3.2.2 Variables of CA

The odds of initial shockable rhythm tended to be higher in 83 patients with CPC 1 and 2 than 105 patients with CPC 3 to 5 (53.0% vs 41.9%, OR = 2.33 [1.20, 4.52], *P* = .01) (Table 3, see Supplemental file 4X, which illustrate forest plot of shockable rhythm in terms of good neurologic outcome predictor).

##### 3.3.2.3 Variables of CPR

**Figure 2:**
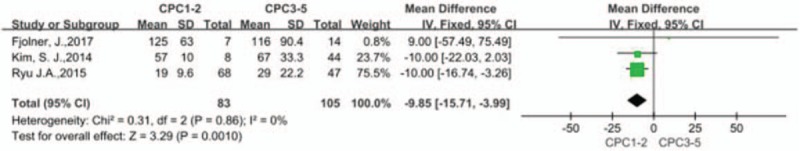
Forest plot of cardiopulmonary resuscitation in terms of good neurologic outcome predictor. CPC = cerebral performance category.

Overall CPR duration tended to be shorter in 83 patients with CPC 1 and 2 than 105 patients with CPC 3 to 5 ([31.6 ± 36.2] minutes vs [56.5 ± 50.6] minutes, MD = −9.85 [−15.71, −3.99] minutes, I < .01) (Table 3, Fig. 2, which illustrates forest plot of CPR in terms of good neurologic outcome predictor). This result was robust in sensitivity analysis (see Supplemental file 4Y, which illustrates forest plot of CPR duration in terms of good neurologic outcome predictor).

##### 3.3.2.4 Quality of evidence

The quality of evidence supporting the prognostic value of all predictors was considered very low. We downgraded 4 points with male and shockable rhythm, and 5 points with age and CPR duration (Table 3, see Supplemental file 5, which illustrates quality of evidence assessment with good neurologic outcome predictors according to GRADE rating system).

## 4 Discussion

This article discussed the predictors for survival and neurologic outcome of ECPR and summarized their characteristics, which were helpful for clinicians making decisions for patient selection at the medical institutions where ECPR was promptly applicable. This meta-analysis extended evidence from previous studies and reported significant associations between survival and IHCA, witnessed CA, bystander CPR, initial shockable rhythm, shorter CPR duration and arrest-to-ECMO duration, higher baseline PH, lower baseline lactate and PCI, at the same time we demonstrated the association between good neurologic outcome and initial shockable rhythm and shorter CPR duration.

Previous research indicated that survival among the elderly supported on ECPR is lower than that for younger adult patients (28.7% vs 40.0%) but higher than that after conventional CPR (17%),^[35]^ suggesting that age should not be a bar against consideration for the use of ECMO in older patients but should be considered on a case-by-case basis.

Compared to patients with OHCA, patients with IHCA could be better candidates for EPCR, and better 30-day and 1-year survival for IHCA patients treated with ECPR than OHCA patients. The difference in outcomes for ECPR after IHCA or OHCA disappeared after adjusting for patient factors and the time delay in starting ECPR.^[12]^ Immediate availability of advanced life support and a better knowledge of the underlying etiologies make the treatment more effective for IHCA patients than OHCA patients.

Just as considered a major favorable predictor for patient with conventional CPR,^[36,37]^ initial shockable rhythm was reported significant trends in our meta-analysis towards survival and good neurologic outcome for patients with ECPR. Though the patients with initial nonshockable rhythm may have a poorer outcome compared with those with initial shockable rhythm, these patients at the time of ECPR could survive. Therefore, a nonshockable rhythm should not be an exclusion criterion for EPCR.

The French guidelines recommend measuring the no-flow time to assess the eligibility of patients for ECPR. The no-flow time is of paramount importance since it is considered as the main variable that determines the neurologic prognosis.^[38]^Conventional CPR achieves 25% of normal cardiac output, and high quality CPR is important to achieve the necessary perfusion to major organs by chest compression.^[39,40]^ Half of IHCA^[2]^ and most OHCA^[3]^ patients achieve ROSC within 10 to 15 minutes, and mostly within 20 minutes. The case that a patient who suffered CA and underwent ECPR after CPR of 30 to 60 minutes surviving with intact neurologic function was reported,^[34]^ indicating that effective CPR before ECPR could supply cerebral perfusion. Witnessed CA and bystander CPR is crucial to survival of patients with EPCR.

The survival and good neurologic outcome probability declines with the CPR duration expanding although ECPR is carried out. Time from CA to ECMO initiation is a critical determinant of outcome with survival rates of 50% when ECMO initiated within 30 minutes of IHCA, 30% between 30 and 60 minutes, and 18% after 60 minutes. Recent evidence showed that the factors most strongly associated with mortality were ongoing CPR at the time of ECMO initiation and arrest to ECMO cannulation time. Interventions aimed at reducing time to ECMO initiation may lead to improved outcomes following ECPR.^[41]^ Recent series suggest that short-term survival can be obtained in 28% to 29.2% of the patients experiencing OHCA, provided that duration from arrest to ECMO is shorter than 60 minutes.^[42,43]^ Longer time interval form collapse to ECMO initiation causes more severe injuries, including the heart and the brain. This would result in difficulties in achieving ROSC. ECMO is used to minimize ischemic injury and to provide protection from cardiac dysfunction and multiple organ failure.^[34,44]^ Previous study illustrated the CPR duration before ECMO application was associated with survival with Modified Rankin scale 0 to 3 (OR 0.95, 95% CI 0.92–0.97).^[45]^ A prolonged low-flow duration is an independent risk of poor neurologic outcome and participates to the multiple organ failure syndrome observed after arrest.^[38]^ CPR duration is an indicator for the implementation of ECPR as well as an index to explain refractoriness to CPR. Providing ECPR to patients with refractory arrest within an optimal CPR duration and arrest-to-ECMO duration is critical to achieve favorable outcomes. If the probability for CA patients ROSC via traditional measures is slim, the clinical decision should be made within 10 minutes and completed within 15 minutes to implement ECPR in the time allowed.^[46]^ Nonetheless, efforts must be made to reduce delays to the initiation of ECPR. Appropriate selection of patients and optimization of organ perfusion during resuscitation may lead to good results in patients with both OHCA and IHCA patients treated with ECPR. Nonetheless, no agreement was reached about the time delay before ECMO pumping on. Substantial between-study heterogeneity in terms of CPR duration observed in this meta-analysis reflected the lack of unified criterion for patient selection of ECPR implementation.

Inadequate tissue oxygenation results in anaerobic metabolism and the development of metabolic acidosis during CPR. Higher baseline arterial PH and lower baseline lactate concentration most likely mirror a longer duration or poor performance of CPR. These routine laboratory data can help clinicians make clinical decisions.

Several other studies have confirmed that PCI may improve survival rate in patient suffered from CA with suspected acute coronary syndrome.^[47,48]^ Blood flow in the myocardium at the distal site of the occluded coronary does not increase even if coronary blood flow is increased by ECMO. Yet PCI could open the occluded artery and reperfuse the distal myocardium. Intra-arrest PCI for ACS patients was effective in achieving high coronary perfusion pressure to restore the heart beat.^[49]^

### 4.1 Limitation

This meta-analysis has a few caveats that must be considered: Firstly, the interpretation of these findings is inevitably limited by the retrospective observational single-centered research, and the quality of evidence is very low. Observational studies are prone to confounding and selection bias in contrast to randomized controlled trials.^[50]^ Substantial between-study heterogeneity and publication bias with primary outcome were observed in our meta-analysis. Systemic review of observational studies may yield precise but spurious factors as consequence. It seems to be that we are not very confident with our reports. Yet the ECPR recipient's prognosis depends on many factors that cannot be randomly assigned, so the findings of observational studies may not be different from that of randomized controlled trails. Secondly, the cases included in this article covered a long span of 23 years from 1994 to 2016. Richardson et al found that despite advances in provision of ECMO care and increasing comorbidities of patients, there has been no change in risk-adjusted survival over time from 2003 to 2014.^[51]^ The long time span would not affect our outcome. Thirdly, this article discussed the predictors for survival and neurologic outcome which was assessed at discharging; however, little was known whether those predictors would play part in long-term clinical benefit. Tae Gun Shin et al reported that age ≤65 years, CPR duration ≤35 minutes and subsequent cardiovascular intervention including coronary intervention or cardiac surgery were associated with 2-year survival,^[52]^ which was partly in correspondence with our outcome. Lastly, despite we discussed several predictors, we still do not know how these predictors play comprehensive roles. The weight of each single variable should be studied further, and an integrated model is needed to be established.

## 5 Conclusion

For adult ECPR recipients, the predictors associated with survival were IHCA, witnessed CA, bystander CPR, initial shockable rhythm, shorter CPR duration and arrest-to-ECMO duration, higher baseline PH, lower baseline lactate and PCI, while initial shockable rhythm and shorter CPR duration were associated with good neurologic outcome. How to use these factors to conduct comprehensive analysis to guide clinical decision-making remains to be further studied.

## Author contributions

**Conceptualization:** Junhong Wang, Qingbian Ma, Yaan Zheng.

**Data curation:** Junhong Wang, Qingbian Ma, Hua Zhang.

**Formal analysis:** Junhong Wang, Hua Zhang, Shaoyu Liu.

**Funding acquisition:** Qingbian Ma, Yaan Zheng.

**Investigation:** Junhong Wang, Shaoyu Liu.

**Methodology:** Junhong Wang, Qingbian Ma, Hua Zhang, Shaoyu Liu.

**Project administration:** Shaoyu Liu.

**Resources:** Junhong Wang, Shaoyu Liu.

**Software:** Junhong Wang, Hua Zhang, Shaoyu Liu.

**Supervision:** Junhong Wang, Qingbian Ma, Hua Zhang, Yaan Zheng.

**Validation:** Junhong Wang, Qingbian Ma, Hua Zhang, Shaoyu Liu, Yaan Zheng.

**Visualization:** Junhong Wang.

**Writing – original draft:** Junhong Wang.

**Writing – review & editing:** Junhong Wang, Qingbian Ma, Yaan Zheng.

## Supplementary Material

Supplemental Digital Content
