# Supplementary material for: Predictors of survival and neurologic outcome for adults with extracorporeal cardiopulmonary resuscitation: A systemic review and meta-analysis
Source: Medicine (Baltimore). 2018 Nov 30;97(48):e13257. doi: 10.1097/MD.0000000000013257 (PMC6283197; doi:10.1097/MD.0000000000013257)
Supplement: Supplemental Digital Content [file medi-97-e13257-s001.doc]

**Search strategies for Pubmed, EMBASE and The Cochrane Library database**

Pubmed (1296) (2018.1.29)

1. “extracorporeal cardiopulmonary resuscitation” [Title/Abstract]253

2. “ECPR” [Title/Abstract]181

3. “extracorporeal membrane oxygenation” [Title/Abstract]7793

4. “ECMO” [Title/Abstract]5599

5. “extracorporeal life support” [Title/Abstract]1541

6. “ECLS” [Title/Abstract]969

7. “extracorporeal membrane oxygenation” [MeSH Terms]7821

8.1 OR 2 OR 3 OR 4 OR 5 OR 6 OR 7 11981

9. “Cardiac arrest” [Title/Abstract] 28177

10. “Heart arrest” [Title/Abstract]2058

11. “Cardiopulmonary resuscitation” [Title/Abstract] 12565

12. “CPR” [Title/Abstract]10290

13. “resuscitation”[Title/Abstract]49448

14. “heart arrest”[MeSH Terms]41953

15. “cardiopulmonary resuscitation”[MeSH Terms]15109

16. “resuscitation”[MeSH Terms]85308

17.9 OR 10 OR 11 OR 12 OR 13 OR 14 OR 15 OR 16 93732

18.“2000/01/01”[Date - Publication] : “3000”[Date - Publication] 14624321

19. “animals” [MeSH Terms] 21268638

20. 18 NOT 19 4179586

21. 8 AND 17 AND 20 1296

Embase（3666）(2018.1.30)

#1 ‘extracorporeal cardiopulmonary resuscitation’:ti,ab,kw

#2 ‘ecpr’ :ti,ab,kw

#3 ‘ecmo’:ti,ab,kw

#4 ‘extracorporeal membrane oxygenation’:ti,ab,kw

#5 ‘extracorporeal life support’:ti,ab,kw

#6 ‘ecls’:ti,ab,kw

#7 ‘extracorporeal oxygenation’/exp

#8 ‘extracorporeal cardiopulmonary resuscitation’/exp

#9 ‘extracorporeal life support’/exp

#10 #1 OR #2 OR #3 OR #4 OR #5 OR #6 OR #7 OR #8 OR #9

#11 ‘cardiac arrest’:ti,ab,kw

#12 ‘heart arrest’:ti,ab,kw

#13 ‘cardiopulmonary resuscitation’:ti,ab,kw

#14 ‘resuscitation’:ti,ab,kw

#15 ‘heart arrest’/exp

#16 ‘resuscitation’/exp

#17 #11 OR #12 OR #13 OR #14 OR #15 #16

#18 ‘human’/exp

#19 [2000-2018]/py

#20 #10 AND #19 19142

#21 #17 AND #19 126377

#22 #18 AND #19 12,229,156

#23 #20 AND #21 3991

#24 #22 AND #23 3666

Cochrane (65) (2018.1.30)

#1 'extracorporeal cardiopulmonary resuscitation':ti,ab,kw (Word variations have been searched)

#2 'extracorporeal membrane oxygenation':ti,ab,kw (Word variations have been searched)

#3 'extracorporeal life support*':ti,ab,kw (Word variations have been searched)

#4 'ecmo':ti,ab,kw (Word variations have been searched)

#5 MeSH descriptor: [Extracorporeal Membrane Oxygenation] explode all trees 200

#6 #1 or #2 or #3 or #4 or #5(Word variations have been searched) 578

#7 'heart arrest':ti,ab,kw (Word variations have been searched)

#8 'cardiac arrest':ti,ab,kw (Word variations have been searched)

#9 'cardiopulmonary resuscitation':ti,ab,kw (Word variations have been searched)

#10 resuscitation*:ti,ab,kw (Word variations have been searched)

#11 MeSH descriptor: [Heart Arrest] explode all trees 1570

#12 #7 or #8 or #9 or #10 or #11 7721

#13 MeSH descriptor: [Human] explode all trees

# 14 #6 and #12 68

#15 #14 and #13 65

Four systemic reviews or meta-analyses

| Author,year | Title | Reference |
| --- | --- | --- |
| D'Arrigo, S., 2017 | Predictors of favourable outcome after in-hospital cardiac arrest treated with extracorporeal cardiopulmonary resuscitation: A systematic review and meta-analysis. | Resuscitation **121**: 62-70 |
| Ortega-Deballon, I., 2016 | Extracorporeal resuscitation for refractory out-of-hospital cardiac arrest in adults: A systematic review of international practices and outcomes. | Resuscitation **101**: 12-20 |
| Cardarelli, M. G., 2009 | Use of extracorporeal membrane oxygenation for adults in cardiac arrest (E-CPR): a meta-analysis of observational studies. | Asaio j **55**(6): 581-586. |
| Debaty, G., 2017 | Prognostic factors for extracorporeal cardiopulmonary resuscitation recipients following out-of-hospital refractory cardiac arrest. A systematic review and meta-analysis. | Resuscitation **112**: 1-10. |

Reasons for exclusion of Trails

| Reason | Author,year | Title | Reference |
| --- | --- | --- | --- |
| 1 | Kagawa, E.,2011. | Should we open the occluded coronary artery in patients with refractory cardiac arrest of acute coronary syndrome using extracorporeal membrane oxygenation and percutaneous coronary intervention? | Eur Heart J 32: 189. |
| 1 | Choi, J. H.,(2012 | Long-term survival and neurological outcome of in-hospital cardiac arrest patients rescued by extracorporeal cardiopulmonary resuscitation. | J Am Coll Cardiol 60: B107. |
| 1 | Kagawa, E.,2012 | Who benefits most from extracorporeal cardiopulmonary resuscitation using extracorporeal membrane oxygenation? | Eur Heart J 33: 636. |
| 1 | Kagawa, E.,2012 | Should we emergently revascularize occluded coronaries for cardiac arrest?: rapid-response extracorporeal membrane oxygenation and intra-arrest percutaneous coronary intervention. | Circulation 126(13): 1605-1613. |
| 1 | Fagnoul, D., 2013 | Extracorporeal life support associated with hypothermia and normoxemia in refractory cardiac arrest. | Resuscitation 84(11): 1519-1524. |
| 1 | Leick, J., 2013 | Door-to-implantation time of extracorporeal life support systems predicts mortality in patients with out-of-hospital cardiac arrest. | Clin Res Cardiol 102(9): 661-669. |
| 1 | Leick, J., 2013 | Extracorporeal life support in patients with in-hospital cardiac arrest. | Eur Heart J 34: 727. |
| 1 | Maekawa, K., 2013 | Extracorporeal cardiopulmonary resuscitation for patients with out-of-hospital cardiac arrest of cardiac origin: a propensity-matched study and predictor analysis. | Crit Care Med 41(5): 1186-1196. |
| 1 | Maekawa, K., 2013 | Refractory ventricular fibrillation and survival in out-ofhospital cardiac arrest treated with extracorporeal cardiopulmonary resuscitation. | Intensive Care Med 39: S304. |
| 1 | Shin, T. G., 2013 | Two-year survival and neurological outcome of in-hospital cardiac arrest patients rescued by extracorporeal cardiopulmonary resuscitation. | Int J Cardiol 168(4): 3424-3430. |
| 1 | Bednarczyk, J. M., 2014 | Resuscitative extracorporeal membrane oxygenation for in hospital cardiac arrest: a Canadian observational experience. | Resuscitation 85(12): 1713-1719. |
| 1 | Mochizuki, K., 2014 | Neurological outcomes after extracorporeal cardiopulmonary resuscitation in patients with out-of-hospital cardiac arrest: a retrospective observational study in a rural tertiary care center. | J Intensive Care 2(1): 33. |
| 1 | Han, S. J., 2015 | Predictors of survival following extracorporeal cardiopulmonary resuscitation in patients with acute myocardial infarction-complicated refractory cardiac arrest in the emergency department: a retrospective study. | J Cardiothorac Surg 10: 23. |
| 1 | Kagawa, E., 2015 | Extracorporeal cardiopulmonary resuscitation for nonshockable rhythms | Circulation 132. |
| 1 | Nakashima, T., 2015 | Sustained ventricular fibrillation during CPR as the strongest predictor for the favorable neurological outcomes in out-of-hospital cardiac arrest patients; the multicenter prospective study of SAVE-J. | Circulation 132. |
| 1 | Pontailler, M., 2015 | Outcomes and predictors of 30-day and long-term mortality in case of cardiopulmonary resuscitation requiring extracorporeal life support in the elderly. | J Heart and Lung Transp 34(4): S227-S228. |
| 1 | Lee, J. J., 2016 | Out-of-hospital cardiac arrest patients treated with cardiopulmonary resuscitation using extracorporeal membrane oxygenation: focus on survival rate and neurologic outcome. | Scand J Trauma Resusc Emerg Med 24: 74. |
| 1 | Mosca, M. S., 2016 | Duration of conventional cardiopulmonary resuscitation prior to extracorporeal cardiopulmonary resuscitation and survival among adult cardiac arrest patients. | Perfusion 31(3): 200-206. |
| 1 | Fujii, T., 2017 | Clinical outcomes in patients with acute hemodynamic collapse supported by extracorporeal life support. | Intern Emerg Med 12(8): 1207-1214. |
| 1 | Jung, C., 2016 | Outcome predictors in cardiopulmonary resuscitation facilitated by extracorporeal membrane oxygenation. | Clin Res Cardiol 105(3): 196-205. |
| 1 | Kuroki, N., 2017 | Association between delay to coronary reperfusion and outcome in patients with acute coronary syndrome undergoing extracorporeal cardiopulmonary resuscitation. | Resuscitation 114: 1-6. |
| 1 | Yukawa, T., 2017 | Neurological outcomes and duration from cardiac arrest to the initiation of extracorporeal membrane oxygenation in patients with out-of-hospital cardiac arrest: a retrospective study | Scand J Trauma Resusc Emerg Med 25(1): 95. |
| 1 | S, I. J., 2018 | Does ecpr improve neurologic outcome for pea with witness? | Crit Care Med 46: 120. |
| 2 | Thiagarajan, R. R., 2009 | Extracorporeal membrane oxygenation to support cardiopulmonary resuscitation in adults. | Ann Thorac Surg 87(3): 778-785. |
| 2 | Avalli, L., 2014 | New treatment bundles improve survival in out-of-hospital cardiac arrest patients: a historical comparison. | Resuscitation 85(9): 1240-1244. |
| 3 | Chen, C. T., 2016 | Prognostic factors for survival outcome after in-hospital cardiac arrest: An observational study of the oriental population in Taiwan. | J Chin Med Assoc 79(1): 11-16. |
| 4 | Stub, D., 2013 | Early experience in refractory cardiac arrest treated with mechanical Cpr, hypothermia, ecmo and early reperfusion (the Cheer Study). | Circulation 128(22). |
| 4 | Schober, A., 2017 | Emergency extracorporeal life support and ongoing resuscitation: a retrospective comparison for refractory out-of-hospital cardiac arrest. | Emerg Med J 34(5): 277-281. |
| 5 | Kagawa, E., 2010 | Assessment of outcomes and differences between in- and out-of-hospital cardiac arrest patients treated with cardiopulmonary resuscitation using extracorporeal life support. | Resuscitation 81(8): 968-973. |
| 5 | Avalli, L., 2011 | Favorable survival of in-hospital compared with out-of-hospital refractory cardiac arrest patients treated with extracorporeal membrane oxygenation: An Italian tertiary care center experience. | Circulation 124(21). |
| 5 | Andvik, S., 2012 | CPR-ECMO for in-hospital cardiac arrest: What are the predictive factors for survival and are we missing anyone? | Heart Lung and Circulation 21: S236. |
| 5 | Hirose, H., 2012 | Prognostic value of venous blood ph levels in patients treated with extracorporeal cardiopulmonary resuscitation after cardiac arrest due to cardiac etiology. | Circulation 126(21). |
| 5 | Schopka, S., 2012 | Extracorporeal life support in cardiopulmonary resuscitation. A single center experience in 103 adults. | Artif Organs 36(5): A39-A40. |
| 5 | Schopka, S., 2012 | Extracorporeal Life Support in cardiopulmonary resuscitation of non-postcardiotomy patients. Single center experience in 103 patients. | Thoracic and Cardiovascular Surgeon 60. |
| 5 | Chen, Y. S., 2013 | Comparison of outcome of extracorporeal cardiopulmonary resuscitation for out-of-hospital and in-hospital cardiac arrest. | Circulation 128(22). |
| 5 | Flörchinger, B.,2013 | NSE-values following ECMO supported resuscitation-a prognostic factor for outcome? | Thoracic and Cardiovascular Surgeon.36(4):266 |
| 5 | Kaneko, M., 2013 | The influence of the ECG pattern in patients treated with ECPR (SOS-KANTO 2012 study interim report). | Crit Care Med 41(12): A117. |
| 5 | Mendiratta, P., 2013 | Cardiopulmonary resuscitation requiring extracorporeal membrane oxygenation in the elderly: a review of the Extracorporeal Life Support Organization registry | Asaio j 59(3): 211-215. |
| 5 | Miki, R., .2013 | Neurological outcome of extracorporeal cardiopulmonary resuscitation for out of hospital cardiac arrests | Eur Heart J 34: 1107. |
| 5 | Jouffroy, R., .2014 | Base excess and lactate as prognostic indicators for patients treated by extra corporeal life support after out hospital cardiac arrest due to acute coronary syndrome. | Resuscitation 85(12): 1764-1768. |
| 5 | Kawashima, T., 2014 | Experience of using venoarterial extracorporeal membrane oxygenation as emergency cardiac support. | Eur Heart J 35: 535. |
| 5 | Sawamoto, K., 2014 | Outcome from severe accidental hypothermia with cardiac arrest resuscitated with extracorporeal cardiopulmonary resuscitation. | Am J Emerg Med 32(4): 320-324. |
| 5 | Wang, C. H., .2014 | Improved outcome of extracorporeal cardiopulmonary resuscitation for out-of-hospital cardiac arrest--a comparison with that for extracorporeal rescue for in-hospital cardiac arrest. | Resuscitation 85(9): 1219-1224. |
| 5 | Gil, E., 2015 | Association of BMI with survival discharge among ECPR patients in in-hospital cardiac arrest. | Crit Care Med 43(12): 52-53. |
| 5 | Hirose, T., 2015 | The relationship between cerebral regional oxygen saturation during extracorporeal cardiopulmonary resuscitation and neurological outcome. | Circulation. |
| 5 | Kuroki, N., 2015 | Prognostic impact of early revascularization in patients with cardiac arrest due to acute coronary syndrome undergoing extracorporeal cardiopulmonary resuscitation | Circulation. |
| 5 | Lin, W. S., 2015 | Improved outcomes of extracorporeal cardiopulmonary resuscitation for out-of-hospital cardiac arrest-a community-wide evaluation | Circulation 96(Suppl 1):18-19 |
| 5 | Stub, D., 2015 | Refractory cardiac arrest treated with mechanical CPR, hypothermia, ECMO and early reperfusion (the CHEER trial) | Resuscitation 86: 88-94. |
| 5 | Chang, W. T., 2016 | Optimal arterial blood O2 and CO2 tensions in the early post-resuscitation phase for cardiac arrest patients receiving extracorporeal cardiopulmonary resuscitation. | Academic Emergency Medicine 23: S204-S205. |
| 5 | Chen, Y. F. and Y. S. Chen,2016 | Is the grey-to-white ratio valuable enough in prediction for cardiac arrest patients rescued with extracorporeal resuscitation? | J Thorac Dis 8(6): E469-E470. |
| 5 | Dennis, M., 2016 | Extracorporeal cardiopulmonary resuscitation for refractory cardiac arrest: a multicentre experience. | [Int J Cardiol.](https://www.ncbi.nlm.nih.gov/pubmed/?term=Extracorporeal+cardiopulmonary+resuscitation+for+refractory+cardiac+arrest:+a+multicentre+experience.) 2017;231:131-136. |
| 5 | Kim, D. H., 2016 | Extracorporeal Cardiopulmonary Resuscitation: Predictors of Survival. | Korean J Thorac Cardiovasc Surg 49(4): 273-279. |
| 5 | Lee, Y. H., 2016 | The prognostic value of the grey-to-white matter ratio in cardiac arrest patients treated with extracorporeal membrane oxygenation. | Resuscitation 99: 50-55. |
| 5 | Lopez-Herce, J. ,2016 | The role of the gray-to-white matter ratio to predict the prognosis of cardiac arrest treated with ECMO. | J Thorac Dis 8(5): 757-761. |
| 5 | Pozzi, M., 2016 | Extracorporeal life support for refractory out-of-hospital cardiac arrest: Should we still fight for? A single-centre, 5-year experience. | Int J Cardiol 204: 70-76. |
| 5 | Sharma, A. S., 2016 | Out-of-hospital cardiac arrest: The prospect of E-CPR in the maastricht region. | Netherlands Heart Journal 24(2): 120-126. |
| 5 | Gil, E., 2017 | Association of body mass index with clinical outcomes for in-hospital cardiac arrest adult patients following extracorporeal cardiopulmonary resuscitation. | PLoS One 12(4): e0176143. |
| 5 | Nishiyama, K., 2017 | Severity assessment of brain damage with rSO2 monitoring for extracorporeal cardiopulmonary resuscitation after out-of-hospital cardiac arrest | European journal of heart failure. Conference: 6th euro-elso annual congress. |
| 5 | Pabst, D. and C. E. Brehm, 2018 | Is pulseless electrical activity a reason to refuse cardiopulmonary resuscitation with ECMO support? | Am J Emerg Med.36(4):637-640 |
| 5 | Pabst, D., 2017 | Predictors of Survival for Nonhighly Selected Patients Undergoing Resuscitation With Extracorporeal Membrane Oxygenation After Cardiac Arrest. | Asaio j.64(3):368-374 |
| 5 | Ryu, J. A., 2017 | The association of findings on brain computed tomography with neurologic outcomes following extracorporeal cardiopulmonary resuscitation. | Crit Care 21(1): 15. |
| 5 | Sato, R., 2018 | Impact of rapid response car system on extracorporeal life support in out-of-hospital cardiac arrest: A retrospective cohort study. | Am J Emerg Med.36(3):442-445 |
| 5 | Takeshige, K., 2018 | The impact of etiology-based differences on extracorporeal cardiopulmonary resuscitation. | Crit Care Med 46: 120. |
| 5 | Yamaoka, A. and N. Bunya, 2018 | Agonal respiration predicts good neurologic outcome in extracorporeal cardiopulmonary resuscitation. | Crit Care Med 46: 115. |
| 6 | Chen, Y. S., 2003 | Analysis and results of prolonged resuscitation in cardiac arrest patients rescued by extracorporeal membrane oxygenation | J Am Coll Cardiol 41(2): 197-203. |
| 6 | Park, S. B., 2013 | Surviving factors of extracorporeal cardiopulmonary resuscitation in adults with in-hospital arrest. | Crit Care Med 41(12): A126. |
| 6 | Huang, L., 2016 | Effect and related factors of extracorporeal cardiopulmonary resuscitation combined with emergent percutaneous coronary intervention on cardiac arrest patients due to acute myocardial infarction. | Chinese Journal of Cardiology 44(7): 570-576. |

1 Did not met endpoint criterion

2 Included children

3 Extracorporeal bypass used

4 Included less than 10 patients

5 Did not report primary outcome

6 Studies included duplicate patients

NOS score of included articles

|  | Selection | | | | Comparability | Exposure | | | total |
| --- | --- | --- | --- | --- | --- | --- | --- | --- | --- |
| First author,  publication year | Case definition adequacy | Representativeness of the cases | Selection of controls | Definition of controls | Comparability of cases and controls | Ascertainment of exposure | Same method | Non-response rate |  |
| Chen, Y. S.,2008 | 1 | 1 | 0 | 1 | 1 | 1 | 1 | 1 | 7 |
| Debaty, G.,2010 | 1 | 1 | 0 | 1 | 0 | 1 | 1 | 1 | 6 |
| Ellouze, O.,2018 | 1 | 1 | 0 | 1 | 1 | 1 | 1 | 1 | 7 |
| Fjolner, J.,2017 | 1 | 1 | 0 | 1 | 2 | 1 | 1 | 1 | 7 |
| Ha, T. S.,2017 | 1 | 1 | 0 | 1 | 2 | 1 | 1 | 1 | 8 |
| Haneya, A.,2012 | 1 | 1 | 0 | 1 | 1 | 1 | 1 | 1 | 7 |
| Huang, L.,2016 | 1 | 1 | 0 | 1 | 1 | 1 | 1 | 1 | 7 |
| Kim, S. J.,2014 | 1 | 1 | 0 | 1 | 1 | 1 | 1 | 1 | 7 |
| Laithier, F. X.,2016 | 1 | 1 | 0 | 1 | 0 | 1 | 1 | 1 | 6 |
| Lee, S. W.,2017 | 1 | 1 | 0 | 1 | 0 | 1 | 1 | 1 | 6 |
| Pang, P. Y. K.,2017 | 1 | 1 | 0 | 1 | 2 | 1 | 1 | 1 | 8 |
| Park, S. B.,2014 | 1 | 1 | 0 | 1 | 2 | 1 | 1 | 1 | 8 |
| Ryu, J. A.,2015 | 1 | 1 | 0 | 1 | 1 | 1 | 1 | 1 | 7 |
| Spangenberg, T.,2016 | 1 | 1 | 0 | 1 | 2 | 1 | 1 | 1 | 8 |
| Truby, L.,2014 | 1 | 1 | 0 | 1 | 1 | 1 | 1 | 1 | 7 |
| Wengenmayer, T.,2017 | 1 | 1 | 0 | 1 | 0 | 1 | 1 | 1 | 6 |

**Forest plot of predictors**


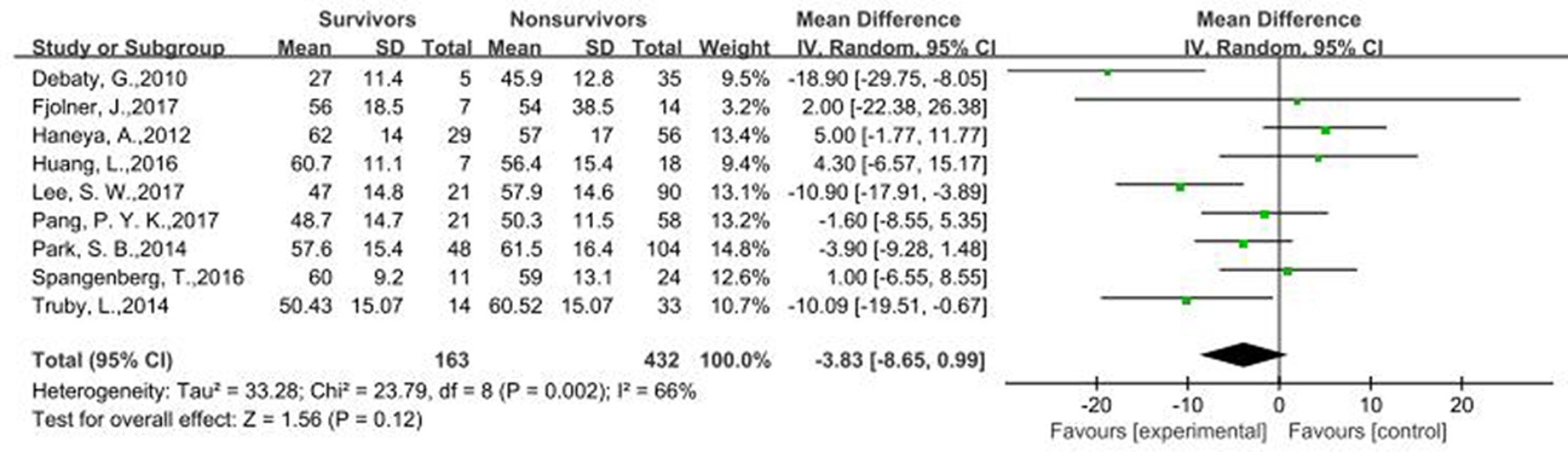


1. Forest plot of age in terms of survival predictor **
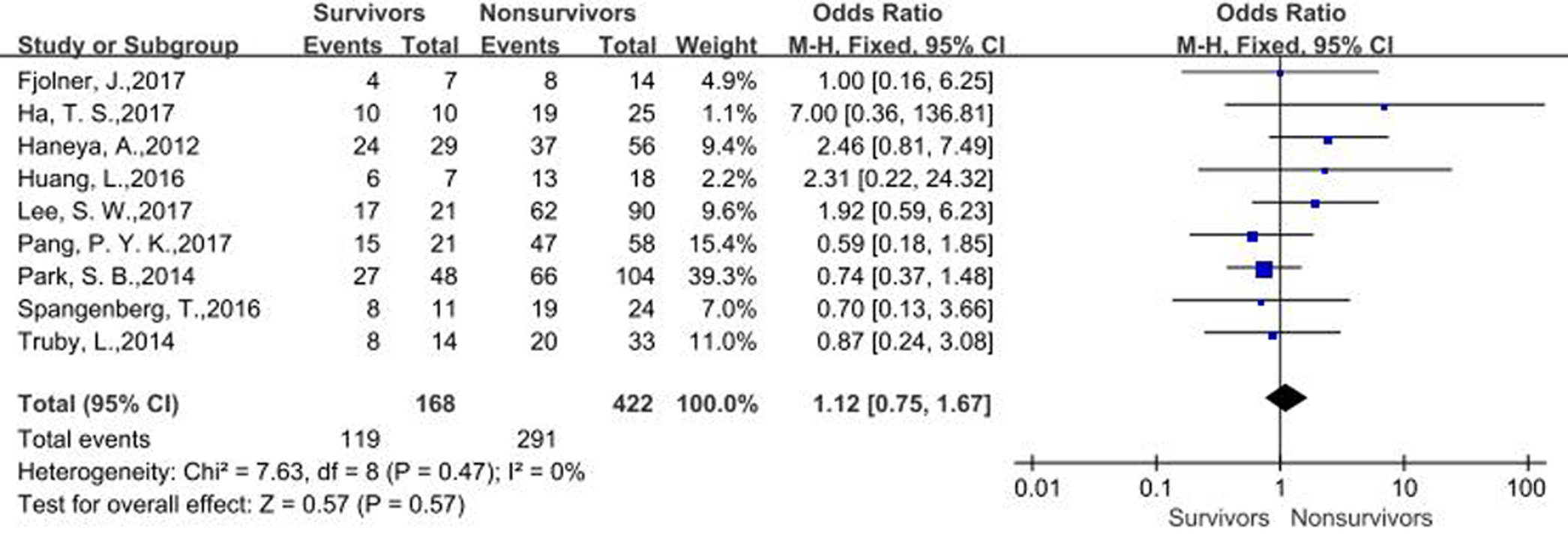
**
2. Forest plot of male in terms of survival predictor


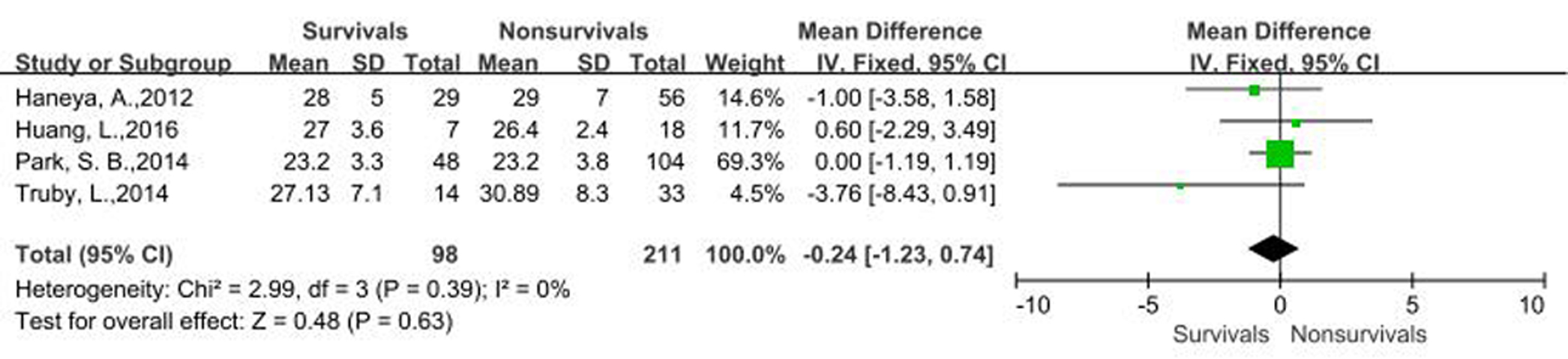


1. Forest plot of BMI in terms of survival predictor


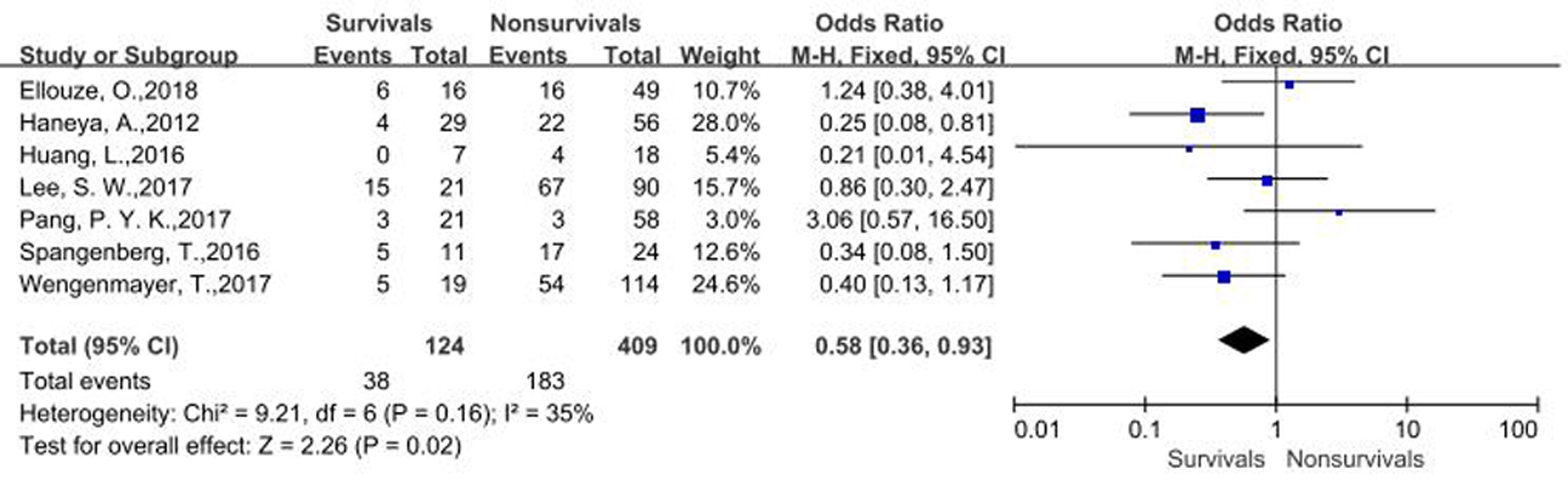


1. Forest plot of OHCA in terms of survival predictor


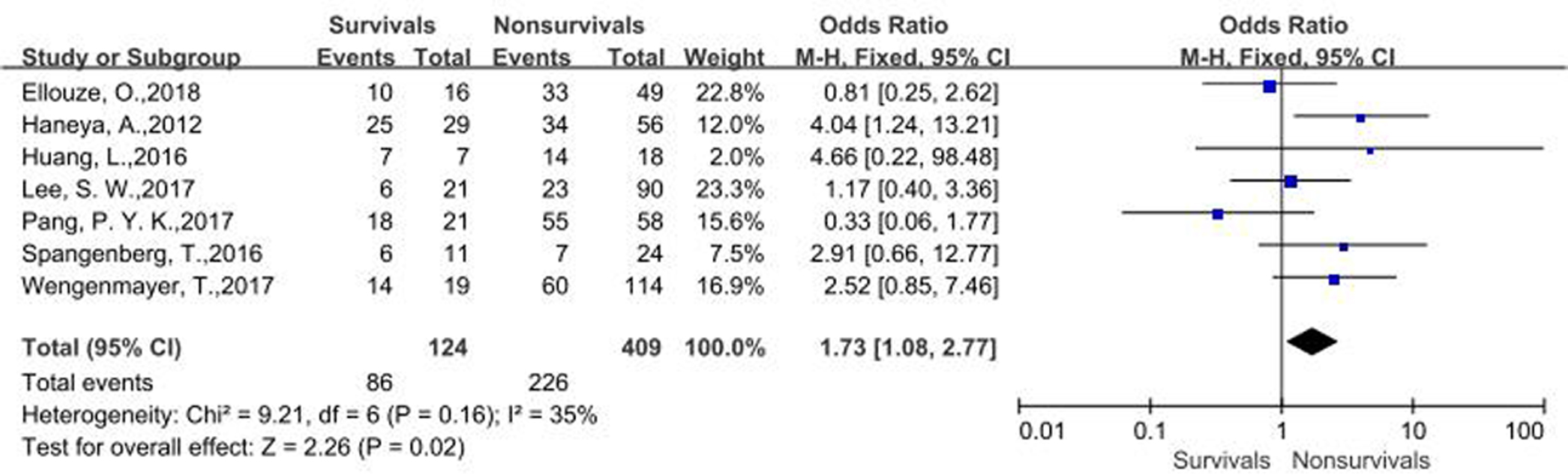


1. Forest plot of IHCA in terms of survival predictor


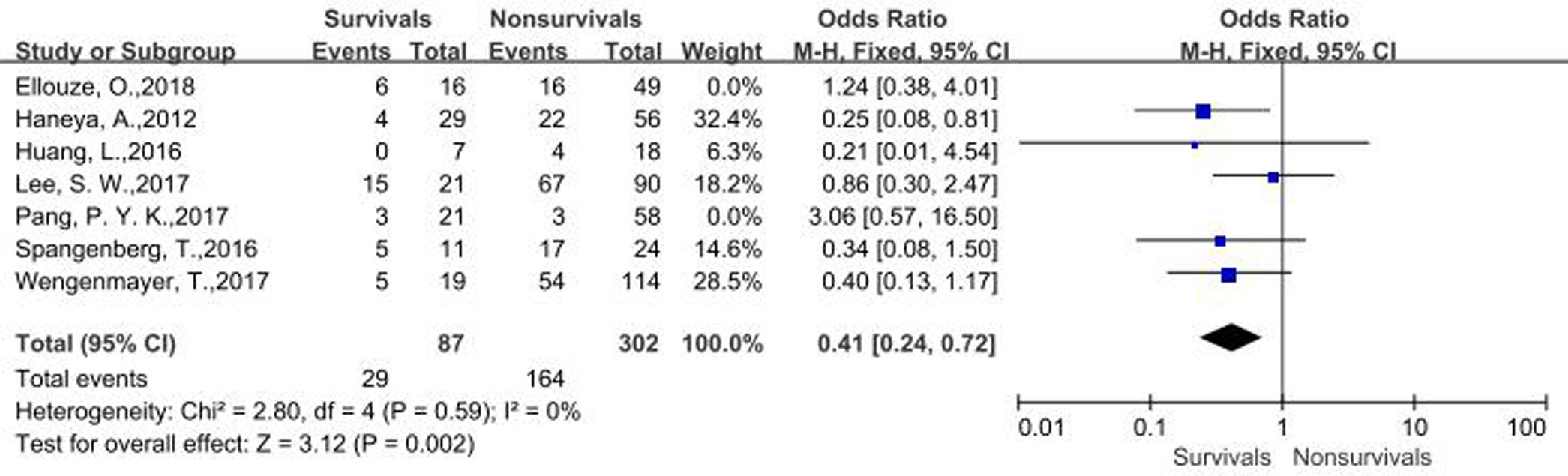


1. Sensitivity of OHCA in terms of survival predictor


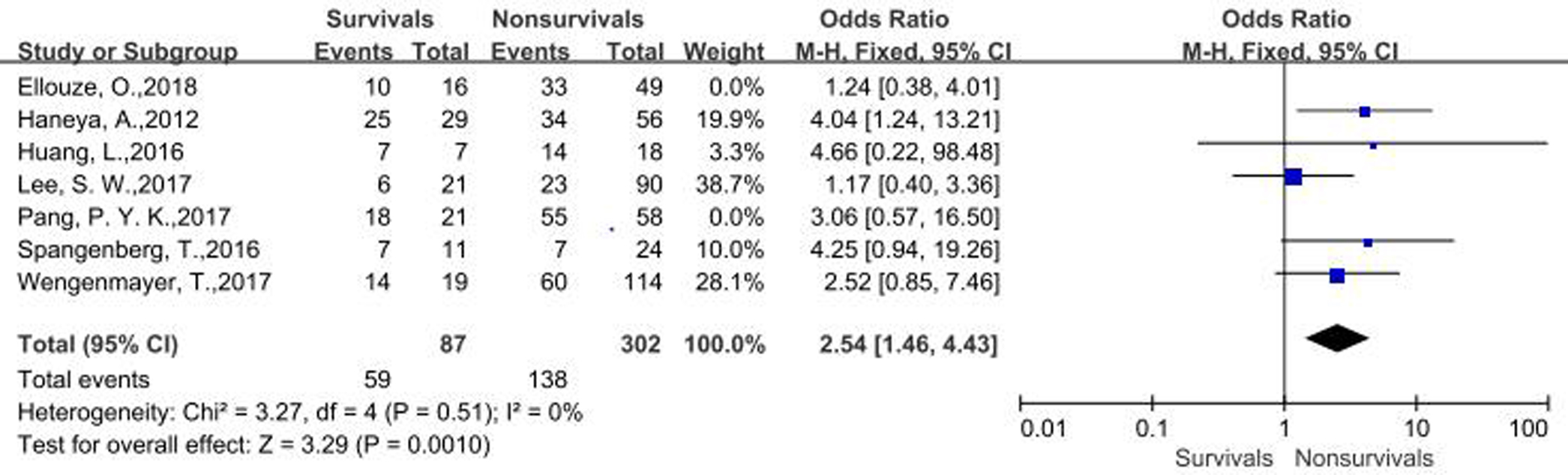


1. Sensitivity of IHCA in terms of survival predictor


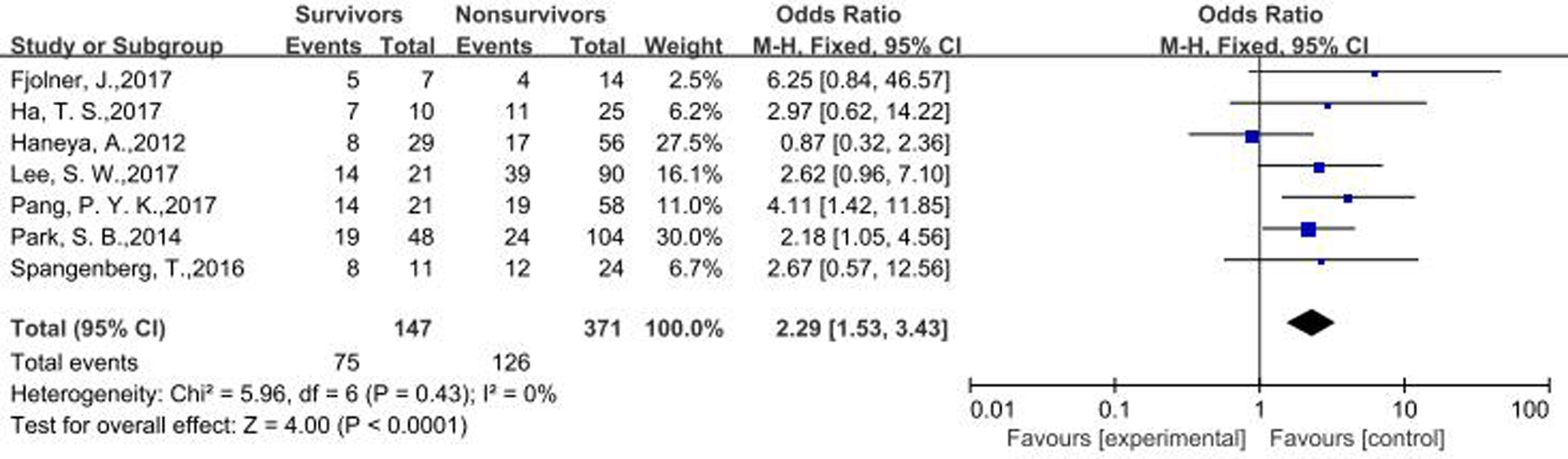


1. Forest plot of initial shockable rhythm in terms of survival predictor


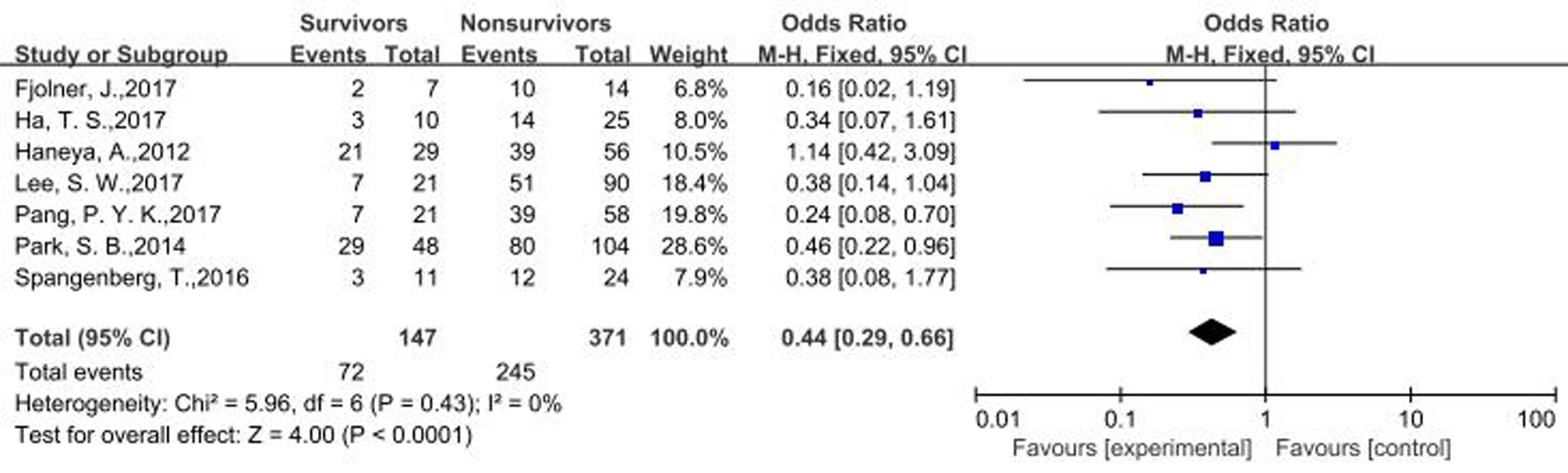


1. Forest plot of initial non-shockable rhythm in terms of survival predictor


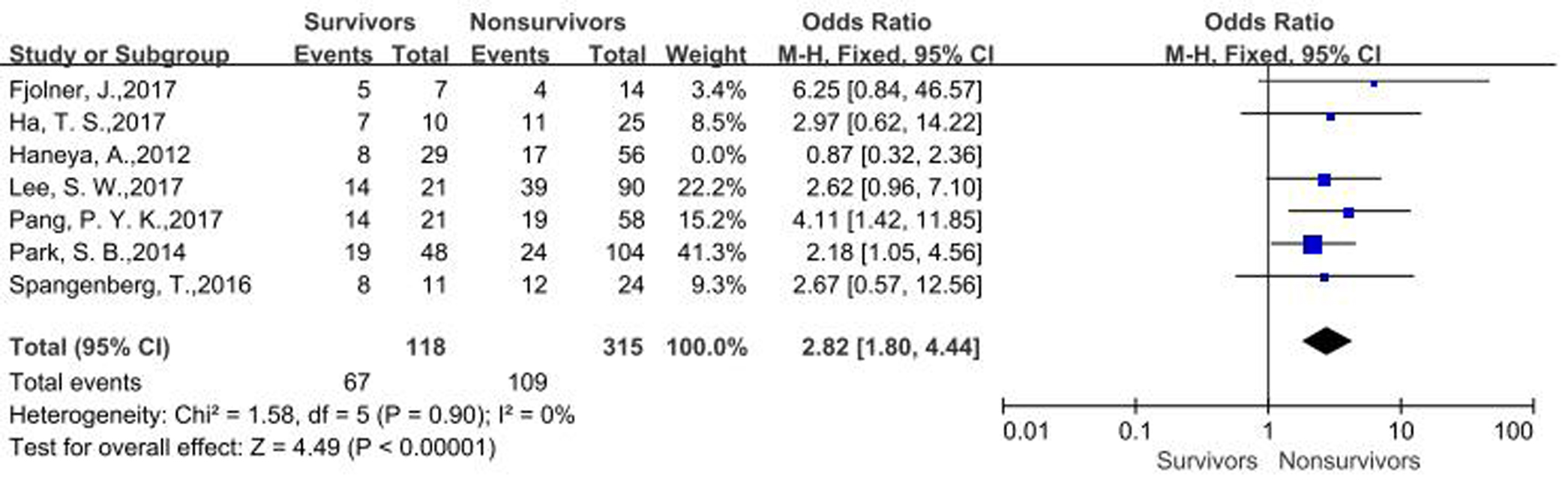


1. Sensitivity analysis of shockable rhythm in terms of survival predictor


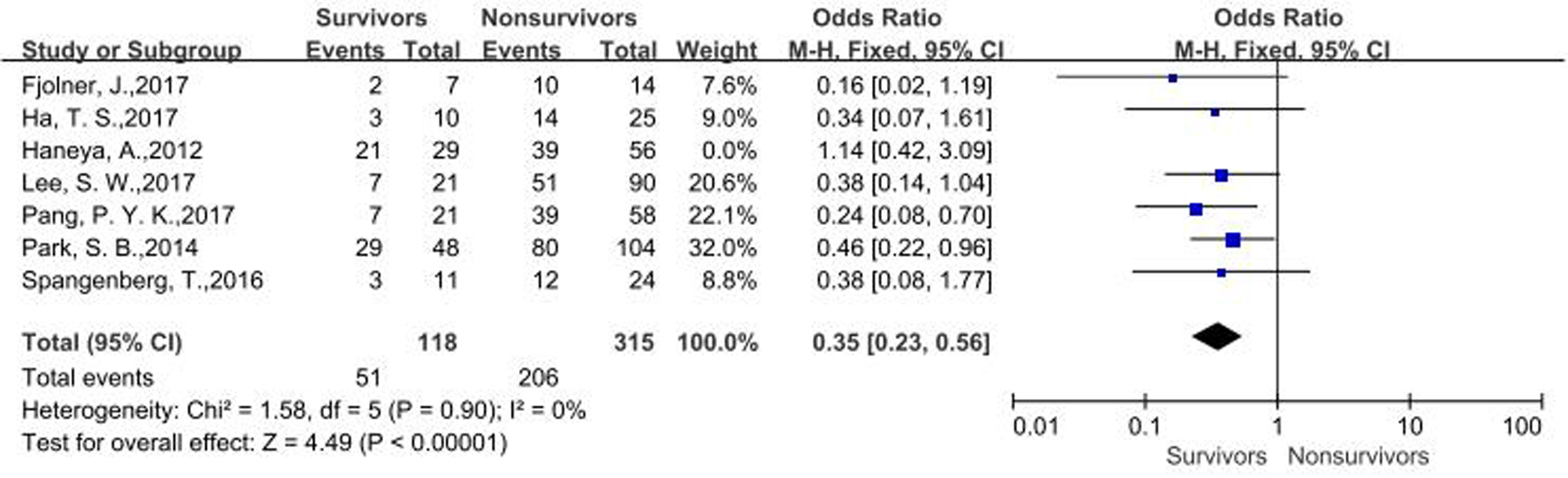


1. Sensitivity analysis of non-shockable rhythm in terms of survival predictor


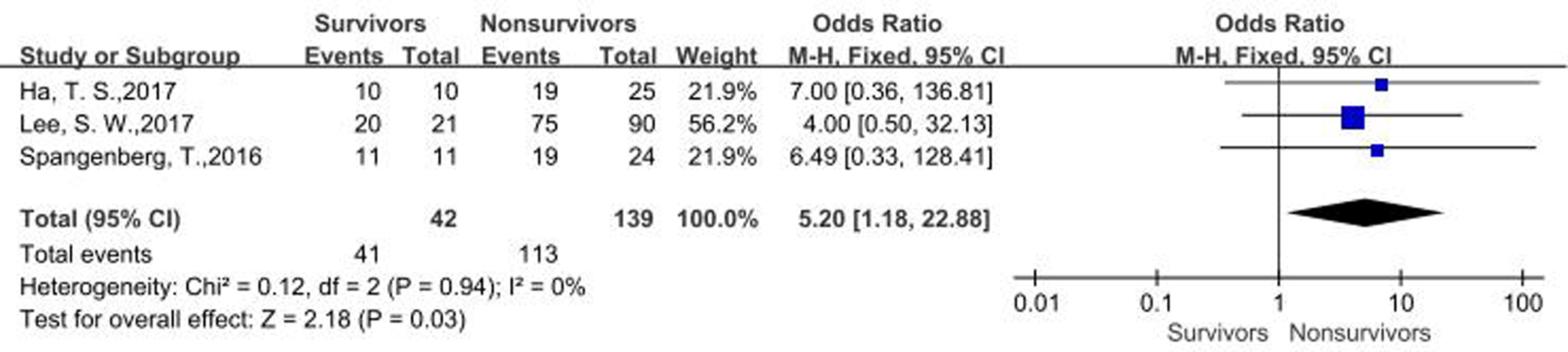


1. Forest plot of witnessed CA in terms of survival predictor


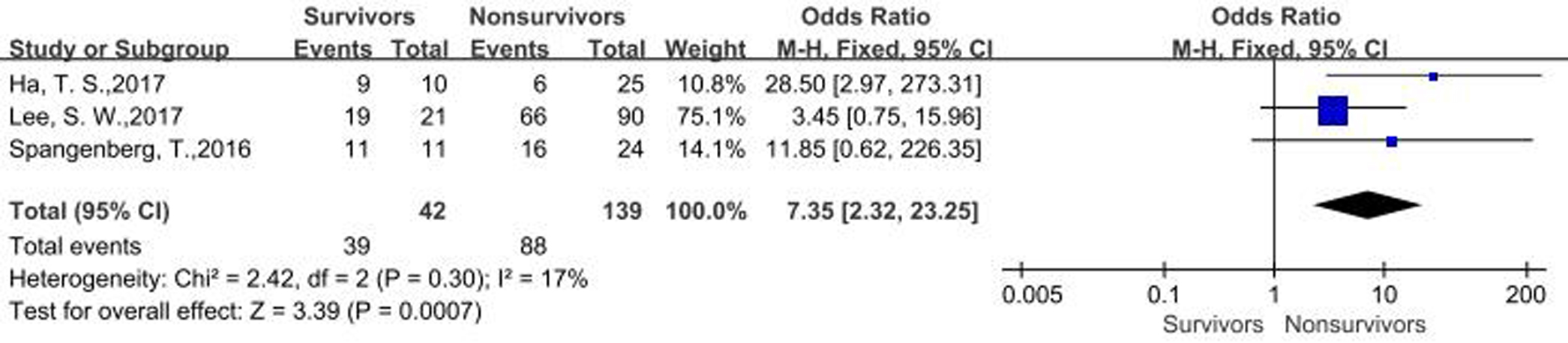


1. Forest plot of bystander CPR in terms of survival predictor


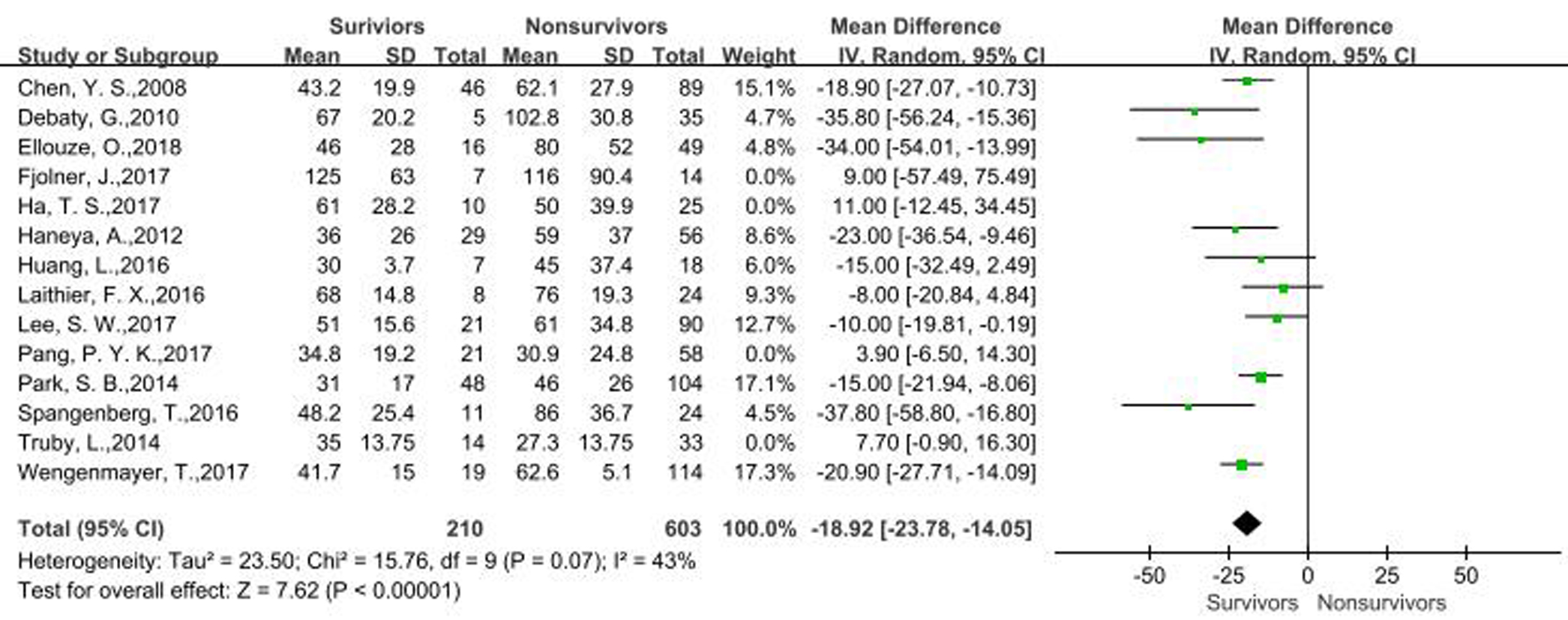


1. Sensitivity analysis of CPR duration in terms of survival predictor


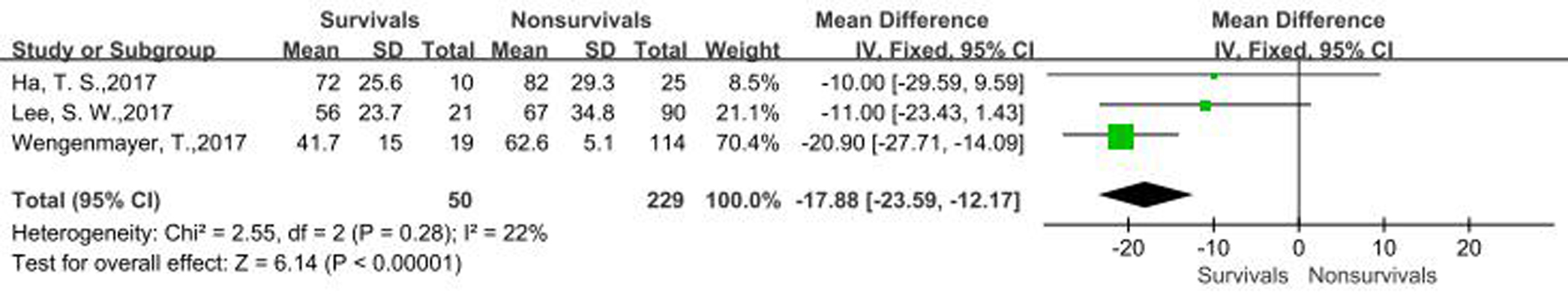


1. Forest plot of arrest-to-ECMO duration in terms of survival predictor


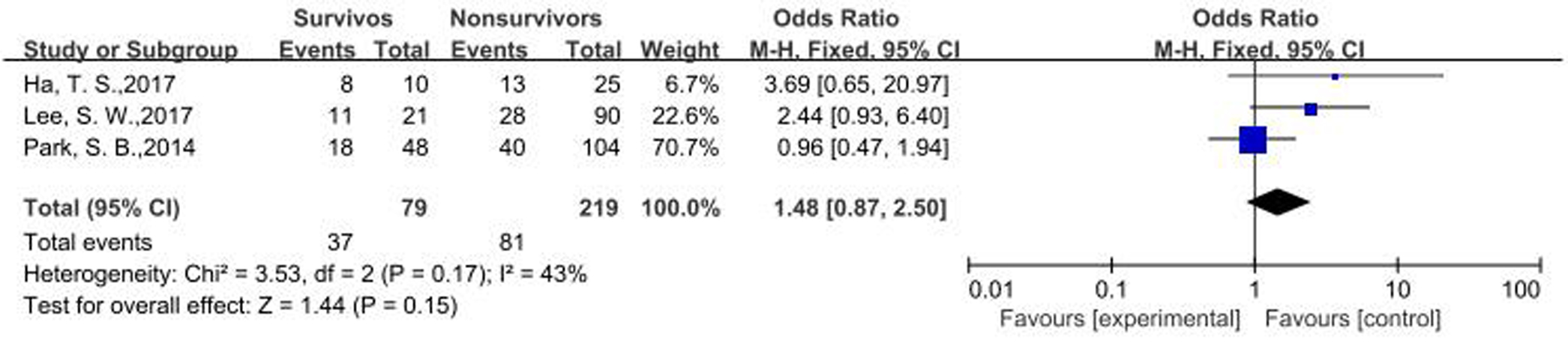


1. Forest plot of ROSC before ECPR in terms of survival predictor


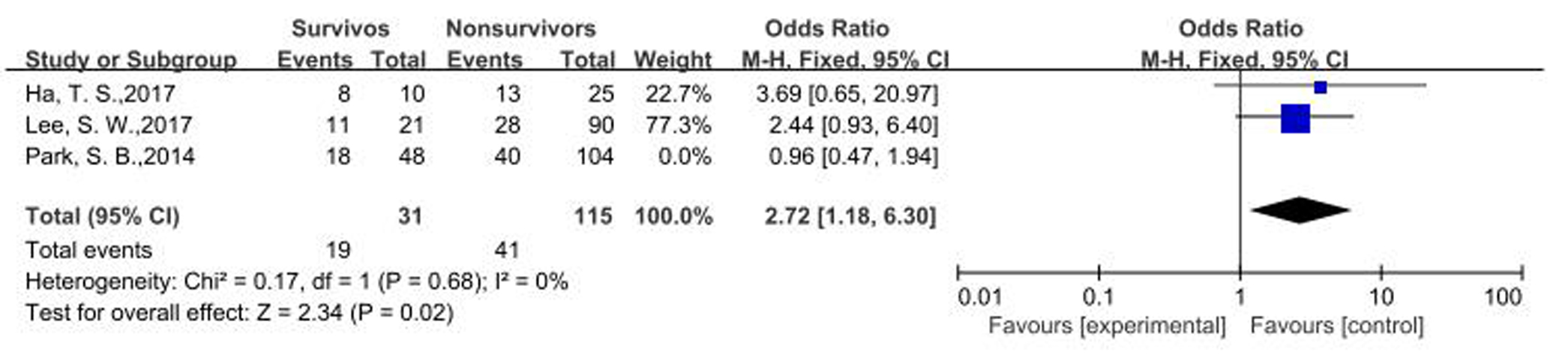


1. Sensitivity analysis of ROSC before ECPR in terms of survival predictor


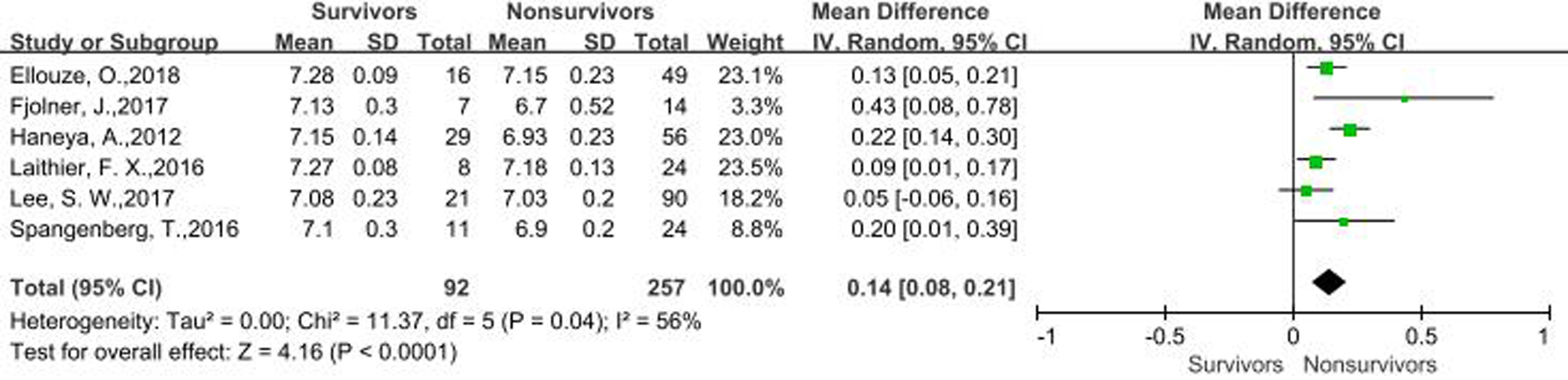


1. Forest plot of baseline PH in terms of survival predictor


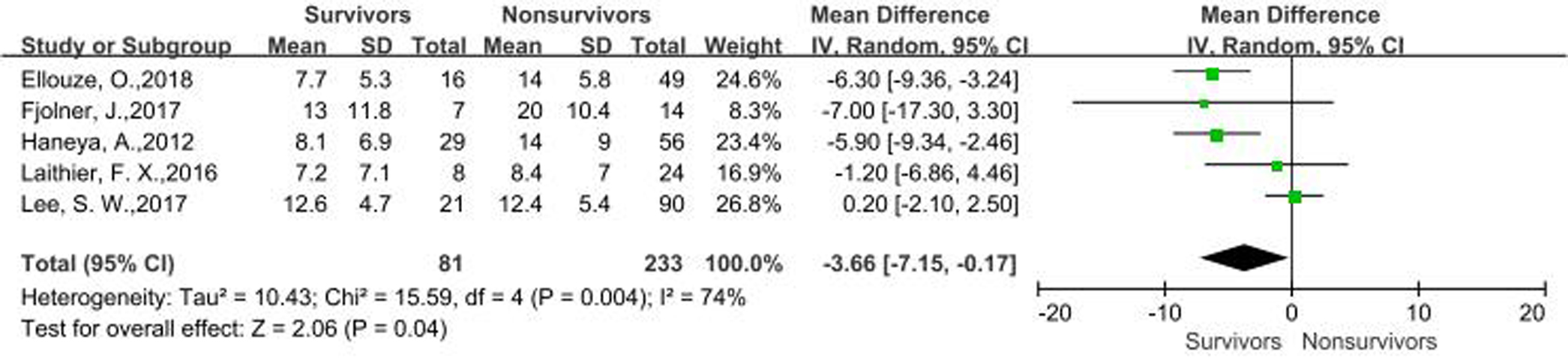


1. Forest plot of baseline lactate in terms of survival predictor


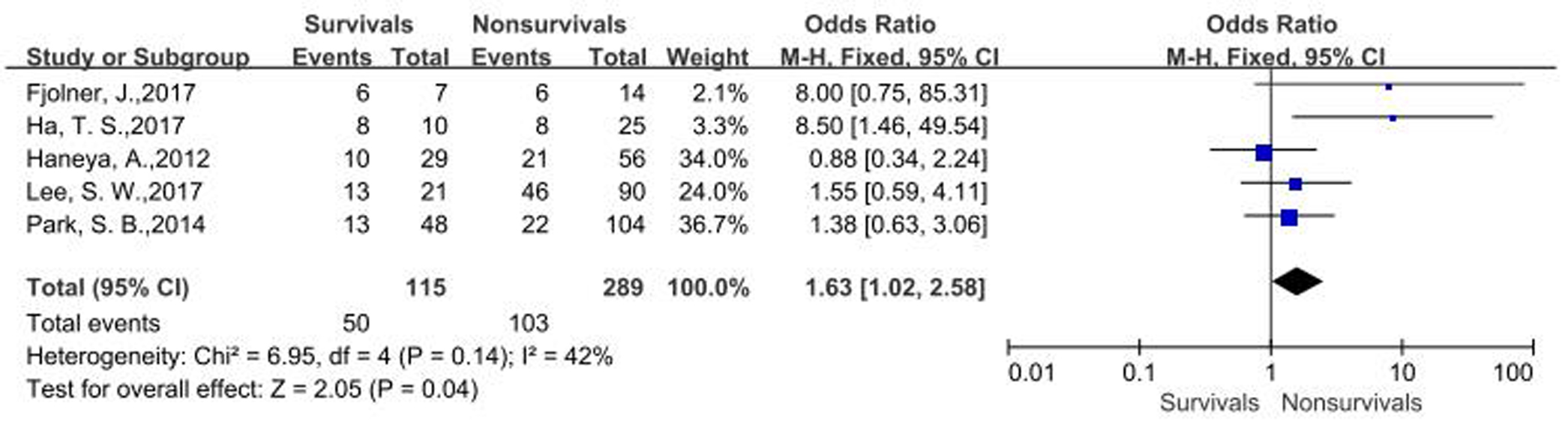


1. Forest plot of PCI in terms of survival predictor


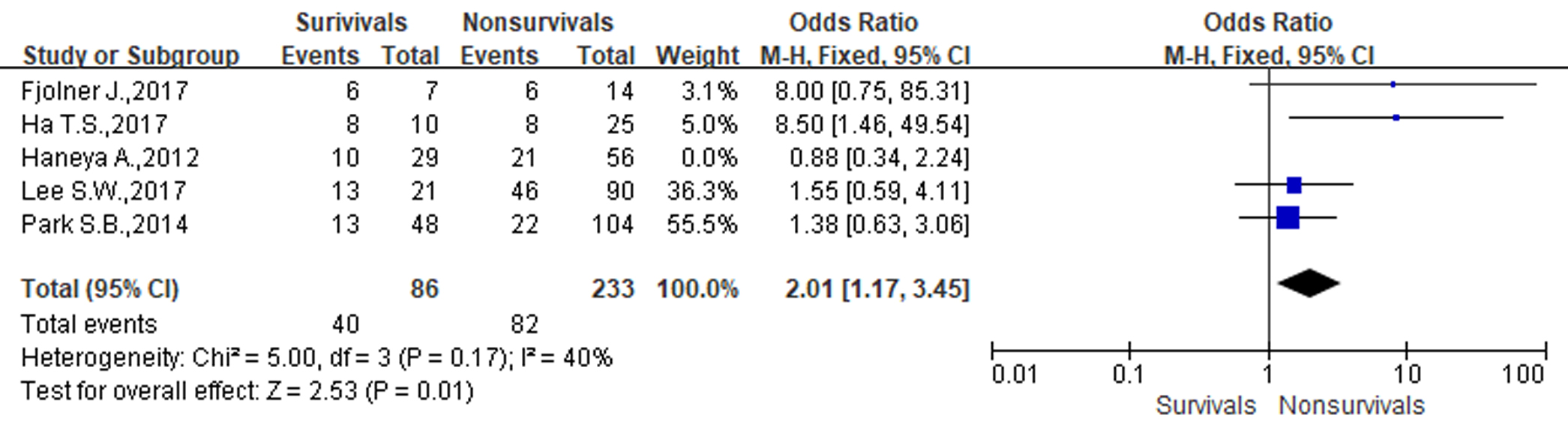


1. Sensitivity analysis of PCI in terms of survival predictor


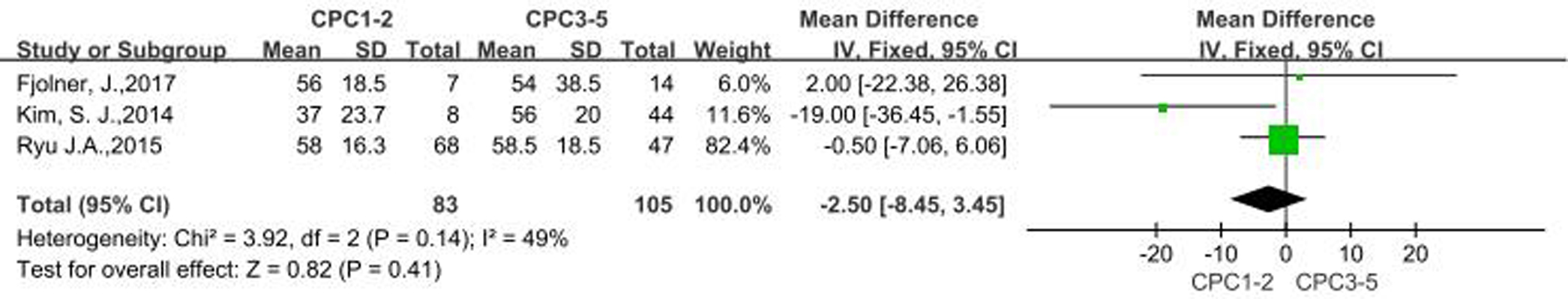


1. Forest plot of age in terms of neurological outcome predictor


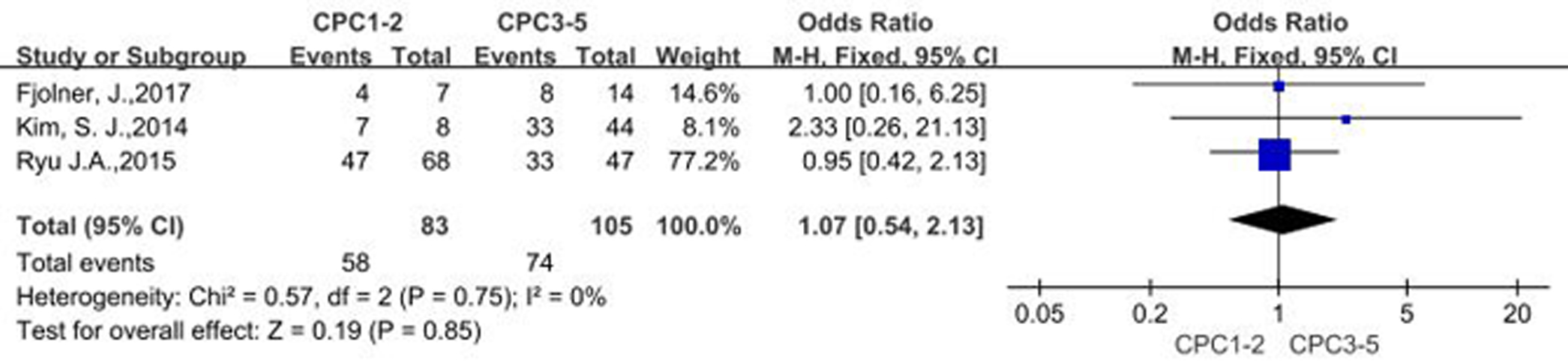


1. Forest plot of male in terms of neurological outcome predictor


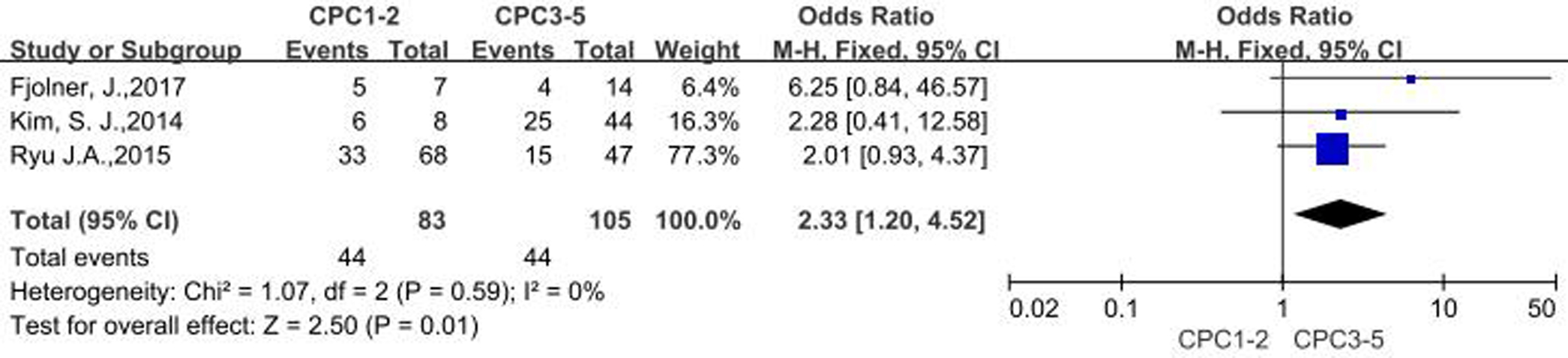


1. Forest plot of shockable rhythm in terms of neurological outcome predictor


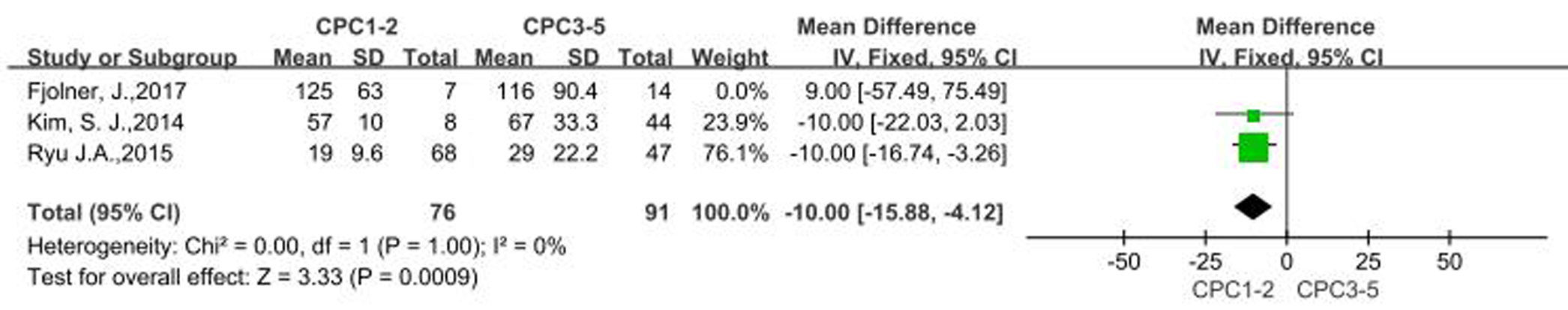


1. Sensitivity analysis of CPR duration in terms of neurological outcome predictor

GRADE score of predictors for discharge

|  | Risk of Bias | Indirectness | Inconsistency | Imprecision | Publication Bias | GRADE |
| --- | --- | --- | --- | --- | --- | --- |
| Age | - | - | - | + - | - | Very low |
| Male | - | + | + | + - | - | Very low |
| BMI | - | + | + | - - |  | Very low |
| IHCA | - | + | + | + - | - | Very low |
| OHCA | - | + | + | - - | - | Very low |
| Shockable rhythm | - | + | + | - - | - | Very low |
| Nonshockable rhythm | - | + | + | + - | - | Very low |
| Witnessed CA | - | - | + | - - | - | Very low |
| Bystander CPR | - | + | + | - - | - | Very low |
| CPR duration | - | - | - | + - | - | Very low |
| Arrest to ECMO | - | - | + | - - | - | Very low |
| ROSC before ECPR | - | + | + | - - | - | Very low |
| PH | - | + | - | - - | - | Very low |
| Lactate | - | + | - | - - | - | Very low |
| PCI | - | + | + | - - | - | Very low |

GRADE score of predictors for neurological outcome

|  | Risk of Bias | Indirectness | Inconsistency | Imprecision | Publication Bias | GRADE |
| --- | --- | --- | --- | --- | --- | --- |
| Age | - | - | + | - - | - | Very low |
| Male | - | + | + | - - | - | Very low |
| Shockable rhythm | - | + | + | - - | - | Very low |
| CPR duration | - | - | + | - - | - | Very low |
